# Efficacy and safety of traditional Chinese medicine in the treatment of osteonecrosis of the femoral head

**DOI:** 10.1186/s13018-023-04086-9

**Published:** 2023-08-14

**Authors:** Wensi Ouyang, Yubo Meng, Guimei Guo, Changwei Zhao, Xiaoling Zhou

**Affiliations:** 1 College of Traditional Chinese Medicine, Changchun University of Chinese Medicine, Changchun, 130117 China; 2 Hospital Affiliated to Changchun University of Traditional Chinese Medicine, Changchun, 130021 China

**Keywords:** Traditional Chinese medicine, Osteonecrosis of the femoral head, Randomized controlled trial, Meta-analysis, Systematic review

## Abstract

**Background:**

Hip joint-preserving treatment options for osteonecrosis of the femoral head (ONFH) have been a research hotspot in recent years. The combination of Chinese and Western medicine has been used in clinical practice to treat early- and mid-stage ONFH. However, there is still a lack of high-quality evidence to verify the effectiveness and safety of this approach.

**Objective:**

To systematically evaluate the clinical efficacy and safety of the combination of traditional Chinese medicine (TCM) with Western medicine in the treatment of early- and mid-stage ONFH.

**Methods:**

Multiple electronic databases were searched to identify the randomized controlled trials (RCTs) examining the use of TCM in the treatment of ONFH. Based on the inclusion and exclusion criteria, eligible studies were selected, and the quality of the studies was evaluated using the risk of bias assessment tool recommended by the Cochrane system Evaluator manual 5.1.0. The meta-analysis of the included data was performed using Review Manager 5.4.1 software and Stata 17.0 software.

**Results:**

A total of 47 RCTs involving 3266 subjects were included in the meta-analysis. The results are observed: (1) Harris score: TCM + Western medicine versus Western medicine (SMD = 1.25, 95% Cl: 1.02 to 1.48, *P* < 0.00001), TCM + physiotherapy versus physiotherapy (SMD = 2.26, 95% Cl: 1.42 to 3.10, *P* < 0.00001), and TCM + hip preservation surgery versus hip preservation surgery (SMD = 1.28, 95% Cl: 1.03 to 1.53, *P* < 0.00001); (2) Visual analogue scale score: TCM + Western medicine versus Western medicine (SMD = −3.99, 95% Cl: −7.41 to −0.57, *P* = 0.02), TCM + physiotherapy versus physiotherapy (SMD = −0.99, 95% Cl: −1.44 to −0.54, *P* < 0.0001), and TCM + hip preservation surgery versus hip preservation surgery (SMD = −1.08, 95% Cl: −1.75 to −0.40, *P* = 0.002); (3) Imaging improvement: TCM + physiotherapy versus physiotherapy (RR = 1.42, 95% Cl: 1.15 to 1.76, *P* = 0.001) and TCM + hip preservation surgery versus hip preservation surgery (RR = 1.21, 95% Cl: 1.11 to 1.31, *P* < 0.0001); and (4) Occurrence of adverse reaction: TCM + Western medicine versus Western medicine (RR = 0.73, 95% Cl: 0.28 to 1.92, *P* = 0.53), TCM + physiotherapy versus physiotherapy (RR = 0.46, 95% Cl: 0.03 to 7.33, *P* = 0.58), and TCM + hip preservation surgery versus hip preservation surgery (RR = 1.11, 95% Cl: 0.36 to 3.45, *P* = 0.86).

**Conclusion:**

TCM combined with Western medicine is an effective and safe approach for the treatment of ONFH. However, due to the low quality and quantity of the included studies, additional large-scale, high-quality studies are required to verify the above conclusions.

Systematic review registration: https://www.crd.york.ac.uk/prospero/#recordDetails, CRD42023392030.

**Supplementary Information:**

The online version contains supplementary material available at 10.1186/s13018-023-04086-9.

## Introduction

Osteonecrosis of the femoral head (ONFH) is a common and challenging disease in orthopedic practice [1]. Most cases are caused by localized ischaemic necrosis of bone tissue caused by glucocorticoid abuse, alcoholism, hip trauma, and other aetiologies, which can lead to microstructural destruction of the femoral head, collapse of the articular surface, hip pain, and functional impairment [2, 3]. At present, the main incidence of ONFH in China is in young and middle-aged people, and the number of new cases of ONFH diagnosed each year is up to approximately two hundred thousand [4, 5]. In addition, the number of new cases in the United States is increasing at a rate of 20,000 each year [6, 7]. This has placed a heavy burden on patients, families, and society. It has also become one of the most important public health issues threatening the health of society [8, 9].

The progression of ONFH often causes severe pain and degenerative changes in the joint. Studies show that more than 80% of untreated ONFH patients will develop femoral head collapse within 1–3 years and eventually have to undergo total hip joint anthropology [10–12]. However, patients may face multiple hip revision surgeries due to factors such as postoperative infection, loosening of instruments, and limitations on the life of the prosthesis [13, 14]. Therefore, early diagnosis and treatment of ONFH are particularly urgent and important. The treatment of ONFH is mainly based on the stage and type of the disease as well as the patient's state. In the early stage, medication, physiotherapy, core decompression, etc., are mainly used to reduce pain, delay the collapse of the femoral head and improve the function of the hip joint, thus achieving the goal of hip preservation [15–17].

A review of domestic and international literature revealed a variety of single Chinese medicines, Chinese medicine monomers, and Chinese medicine compounds can alleviate the main pathological changes in ONFH by regulating bone metabolism, lipid metabolism, and oxidative stress [18, 19]. Traditional Chinese medicine (TCM) has multiple components, targets, and pathways [20]. In recent years, numerous clinical trials have shown that combined TCM therapy can increase efficacy, reduce adverse effects, and delay the process of ONFH, thus playing an active role in the early repair of the disease and improving immunity and quality of life for patients after hip preservation surgery [21, 22]. Therefore, this approach has become highly recommended in treatment guidelines and expert consensus. Nevertheless, there is a lack of high-quality evidence to support the clinical efficacy of TCM in the treatment of ONFH. Hence, this study aims to evaluate the efficacy and safety of TCM in the treatment of ONFH using an evidence-based approach and to seek a clinical basis for combining Chinese and Western medicine in the treatment of early- and mid-stage ONFH.

## Methods and materials

### Protocol Resiter

This systematic review and meta-analysis were conducted in accordance with the Cochrane Handbook of Systematic Reviews and reported in accordance with the Preferred Reporting Items for Systematic Reviews and Meta-Analyses [23, 24]. This meta-analysis was registered with the PROSPERO platform (CRD42023392030).

### Search strategy

The PubMed, Web of Science, Embase, Cochrane Central Register of Controlled Trials, Chinese National Knowledge Infrastructure, China Science and Technology Journal Database, WanFang, and Chinese Biological Medicine electronic databases were comprehensively searched from inception to February 28, 2023. The keywords included “traditional Chinese medicine”, “Chinese medicinal herbs”, “pill”, “decoction”, “capsule”, “osteonecrosis of the femoral head”, “femur head necrosis”, “ONFH”, “FHN”. Searches were conducted using a combination of theme and free words and adapted to the characteristics of each database. In addition, we reviewed the reference lists of included articles for other eligible studies. Only articles in English and Chinese were considered. The detailed search strategy for PubMed was as follows: ((((traditional Chinese medicine [Title/Abstract]) OR (Chinese medicinal herbs [Title/Abstract]) OR (pill [Title/Abstract])) OR (decoction [Title/Abstract])) OR (capsule [Title/Abstract]) AND ("osteonecrosis of the femoral head" [Mesh]) OR ((femur head necrosis [Title/Abstract])) OR (ONFH [Title/Abstract])) OR (FHN [Title/Abstract]). The detailed search strategy used is described in Additional file 1.

### Inclusion criteria

1. Type of studies: Only randomized controlled trials (RCTs) related to the use of TCM for ONFH were included.
2. Type of participants: Patients who met the diagnostic criteria of ONFH. Patients were not limited by age, sex, or race. Diagnostic criteria and staging refer to the Association Research Circulation Osseous 0-III stage and Ficat 0-III stage [25, 26].
3. Type of interventions: The control group was treated with conventional Western medical treatment as prescribed in the guidelines, including anticoagulants, lipid-regulating drugs, osteoclast inhibition and increased osteogenesis, and hip preservation surgery. The treatment group was treated with traditional Chinese herbal medicine combined with the control group.
4. Type of outcomes measures: The primary outcomes included the Harris score, visual analogue scale score, and imaging improvement. The additional outcome was an occurrence of adverse reaction.

### Exclusion criteria

1. RCTs with similar data and multiple publications.
2. Literature reviews, case reports, basic experimental studies, empirical summaries, etc.
3. Data recorded in the literature are unknown.
4. There were no primary or relevant outcome indicators in the RCTs.
5. A study of the use of Chinese medicine therapies other than oral Chinese medicine in interventions.

### Data extraction

Two researchers independently screened the literature for inclusion criteria and exclusion criteria. Disagreements were resolved by discussion or consulting a third researcher. The following data were extracted: study title, publication year, first author, number of cases, interventions, duration of treatment, and outcome indicators.

### Assessment of literature quality

Two reviewers independently evaluated the methodological quality of each included study using the Cochrane Risk of Bias tool, which assessed the following characteristics: random sequence generation, allocation concealment, blinding, incomplete result data, selective reporting, and other biases [27].

### Statistical analysis

Review Manager 5.4.1 software (Cochrane Collaboration, Oxford, UK) and Stata 17.0 software (StataCorp, College Station, USA) were used for statistical analysis. Risk ratio (RR) was used for binomial variables, and standardized mean difference (SMD) was used for continuous variables, both with 95% confidence interval (CI) to describe the effect value of the treatment groups and control groups comparison. Due to differences in the treatment duration and herbal compositions, there was significant clinical heterogeneity in the included studies. So, regardless of statistical heterogeneity, we would use a random effects model to analyze the data. To test the robustness of the outcome, a sensitivity analysis was performed by removing included studies one by one. If an article was excluded and the result was reversed, the article would be shown to be a source of heterogeneity and the article will be analyzed in depth. Otherwise, the results were robust. When the number of included articles was more than 10, the underlying publication bias was identified via an informal visual examination of a funnel plot. Publication bias was evaluated with Egger’s test.

## Results

### Study selection

**Fig. 1:**
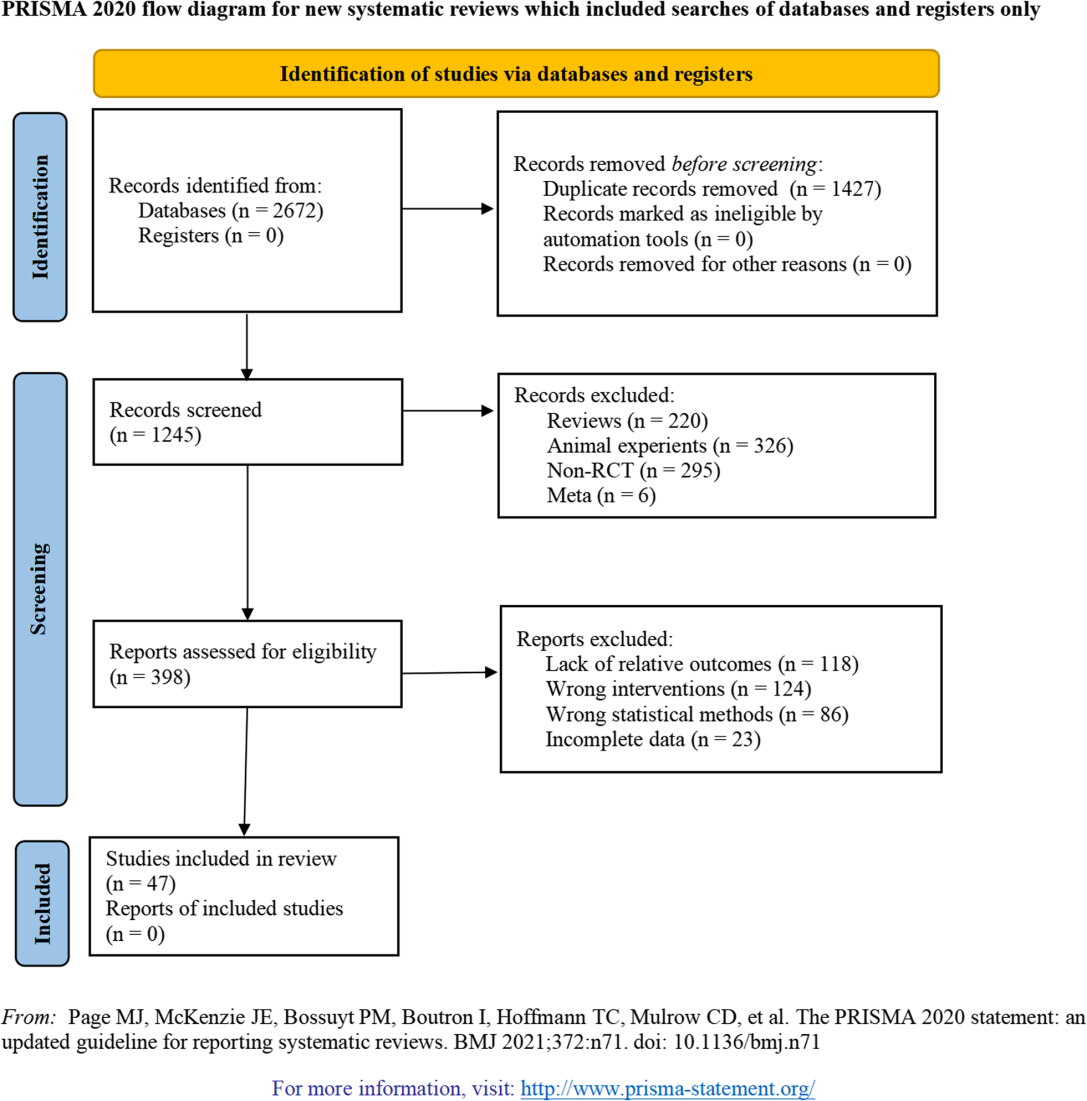
Literature screening process and results

A total of 2672 potentially relevant articles on the treatment of TCM by ONFH were preliminarily retrieved from the databases, and 1427 duplicate records were removed. A total of 847 articles were excluded by reviewing the titles and abstracts. A total of 351 articles were eliminated after reading the full texts and applying the inclusion and exclusion criteria. As a result, a total of 47 published articles [28–74] were ultimately included in the meta-analysis (Fig. 1).

### Research characteristics

A total of 3266 adult participants with ONFH were included in the 47 eligible studies [28–74]. There were 1624 participants in the control group and 1642 participants in the treatment group. All studies had clear inclusion and exclusion criteria, and there were no significant differences in baseline information between the control groups and treatment groups. The control group intervention in all studies was Western medicine alone. The treatment group interventions in all studies were oral Chinese medicine combined with Western medicine (Tables 1, 2). The traditional Chinese herbal medicines commonly used in the treatment group were Danshen (Radix Salviae Miltiorrhizae), Niuxi (Radix Achyranthis Bidentatae), and Huangqi (Radix Astragali). The detailed physiotherapy protocols and traditional Chinese herbal medicine composition are presented in Additional file 1: Table S1. Nineteen studies [31, 32, 35, 38–40, 42–45, 49, 50, 54, 56, 58, 61, 68, 70, 74] were supported by the government or a professional organization, and twenty-eight studies [28–30, 33, 34, 36, 37, 41, 46–48, 51–53, 55, 57, 59, 60, 62–67, 69, 71–73] did not report the funding. Five studies [30, 45, 50, 53, 64] included treatment group interventions that involved TCM combined with Western medicine. Eleven studies [29, 32–34, 36, 38–40, 44, 49, 51] included treatment group interventions that involved TCM combined with physiotherapy (Additional file 1: Table S2). Thirty-one studies [28, 31, 35, 37, 41–43, 46–48, 52, 54–63, 65–74] included a treatment group intervention that involved TCM combined with hip preservation surgery.

**Table 1:** Basic characteristics of the 47 studies included in the meta-analysis

Inclusion studies	Original place of patients	Sample (M/F)	Age (years)	Diagnostic standard	Disease stage
Zheng 2022 [28]	Zhejiang	T: 46 (28/18) C: 46 (26/20)	T: 36.03 ± 5.11 C: 35.58 ± 5.08	ARCO	II/III
Zhang 2022 [29]	Tianjin	T: 42 (17/25) C: 42 (16/26)	T: 41.21 ± 4.33 C: 41.25 ± 4.37	ARCO	I
Shen 2022 [30]	Heilongjiang	T: 46 (30/16) C: 46 (32/14)	T: 56.34 ± 5.29 C: 56.05 ± 5.11	ARCO	II
Sun 2022 [31]	Henan	T: 28 (15/13) C: 30 (17/13)	T: 40.64 ± 10.79 C: 38.10 ± 10.69	ARCO	II/III
Li 2022 [32]	Heilongjiang	T: 36 (20/16) C: 36 (22/14)	T: 43.6 ± 5.1 C: 42.7 ± 6.8	ARCO	I/II
Han 2021 [33]	Anghui	T: 30 (16/14) C: 30 (17/13)	T: 42.19 ± 3.58 C: 42.32 ± 3.92	ARCO	I/II
Liao 2021 [34]	Hunan	T: 22 (13/9) C: 21 (12/9)	T: 44.17 ± 6.97 C: 42.76 ± 5.03	ARCO	I/II
Han 2021 [35]	Hebei	T: 24 (16/14) C: 24 (12/12)	T: 42.19 ± 3.58 C: 42.32 ± 3.92	ARCO	I/II
Liao 2021 [36]	Hunan	T: 44 (27/17) C: 42 (24/18)	T: 43.7 ± 6.9 C: 42.3 ± 5.1	ARCO	I/II
Sun 2021 [37]	Guangxi	T: 16 (8/8) C: 16 (9/7)	T: 37.56 ± 9.85 C: 38.62 ± 8.10	ARCO	I/II
Liu 2020 [38]	Shanxi	T: 50 (28/22) C: 50 (27/23)	T: 65.13 ± 4.58 C: 65.25 ± 4.76	Ficat	I/II/III
Du 2020 [39]	Henan	T: 40 (28/12) C: 40 (31/9)	T: 43.12 ± 7.38 C: 44.73 ± 5.81	ARCO	I/II
Zhao 2020 [40]	Shandong	T: 17 (NA) C: 14 (NA)	T: 37 C: 35	ARCO	I/II/III
Yan 2020 [41]	Henan	T: 43 (33/10) C: 43 (31/12)	T: 38.2 ± 7.9 C: 39.4 ± 8.1	ARCO	I/II
Wei 2019 [42]	Nanjing	T: 16 (9/7) C: 14 (8/4)	T: 34.5 ± 6.5 C: 34.4 ± 6.4	ARCO	III
Sun 2019 [43]	Guangzhou	T: 29 (25/4) C: 31 (26/5)	T: 43.6 ± 4.8 C: 44.7 ± 5.3	ARCO	I/II
Zhou 2019 [44]	Henan	T: 30 (24/6) C: 30 (22/8)	T: 54.20 ± 5.68 C: 55.93 ± 6.92	ARCO	I/II
Zhan 2019 [45]	Zhejiang	T: 41 (NA) C: 41 (NA)	T: 43.52 ± 7.94 C: 42.81 ± 9.07	ARCO	I/II
Zhao 2019 [46]	Shandong	T: 21 (17/4) C: 21 (15/6)	T: 39.29 ± 7.99 C: 37.43 ± 7.14	ARCO	I/II/III
Cao 2018 [47]	Henan	T: 30 (16/14) C: 30 (15/15)	T: 40.53 ± 9.79 C: 41.13 ± 9.64	ARCO	I/II
Wang 2018 [48]	Shanxi	T: 46 (27/19) C: 46 (25/21)	T: 46.2 ± 6.53 C: 45.82 ± 6.37	Ficat	I/II
Song 2018 [49]	Henan	T: 30 (17/13) C: 30 (15/15)	T: 32.64 ± 8.65 C: 32.65 ± 8.76	ARCO	I/II
Du 2018 [50]	Henan	T: 36 (NA) C: 34 (NA)	T: 43.12 ± 7.38 C: 44.73 ± 5.81	ARCO	I/II
Yuan 2018 [51]	Guangzhou	T: 34 (NA) C: 34 (NA)	T: 30.9 ± 6.3 C: 31.5 ± 5.9	ARCO	I/II
Liu 2017 [52]	Shandong	T: 42 (NA) C: 40 (NA)	T: 39.8 C: 39.8	Ficat	I/II
Lu 2017 [53]	Zhejiang	T: 40 (NA) C: 40 (NA)	T: 48.6 ± 4.9 C: 48.6 ± 4.9	Ficat	I/II
Jiang 2017 [54]	Henan	T: 27 (21/6) C: 27 (19/8)	T: 41.66 ± 11.39 C: 40.55 ± 12.81	ARCO	I/II
Li 2017 [55]	Shanxi	T: 51 (38/13) C: 51 (37/14)	T: 45.12 ± 6.77 C: 45.71 ± 7.25	ARCO	I/II
He 2017 [56]	Guangxi	T: 30 (NA) C: 30 (NA)	T: 38.9 ± 9.21 C: 39.25 ± 10.12	ARCO	0/I/II
Xu 2017 [57]	Shanxi	T: 41 (39/12) C: 42 (32/10)	T: 37.01 ± 8.23 C: 36.92 ± 7.51	ARCO	I/II
Zhang 2016 [58]	Jiangsu	T: 32 (24/8) C:29 (22/7)	T: 36.30 ± 9.65 C: 35.27 ± 9.36	ARCO	II/III
Zhang 2016 [59]	Shanxi	T: 36 (NA) C:36 (NA)	T: NA C: NA	ARCO	II/III
Nong 2016 [60]	Guangxi	T: 44 (NA) C:43 (NA)	T: 40.7 ± 10.5 C: 40.7 ± 10.5	Ficat	I/II
Tian 2016 [61]	Henan	T: 25 (14/11) C: 25 (13/12)	T: 36 ± 7 C: 36 ± 6	ARCO	I/II/III
Zhu 2015 [62]	Zhejiang	T: 33 (NA) C: 33 (NA)	T: 38.27 ± 4.06 C: 38.27 ± 4.06	ARCO	II/III
Liu 2015 [63]	Sichuan	T: 39 (26/13) C: 39 (25/14)	T: 39.82 ± 6.83 C: 39.90 ± 6.74	Ficat	0/I/II
Zhou 2015 [64]	Hebei	T: 52 (18/34) C: 53 (17/36)	T: 51.45 ± 5.41 C: 51.33 ± 5.27	ARCO	I/II
Wang 2015 [65]	Liaoning	T: 47 (28/19) C: 47 (29/18)	T: 35.4 ± 11.3 C: 34.5 ± 10.6	Ficat	I/II
Li 2015 [66]	Hebei	T: 35 (24/11) C: 33 (23/10)	T: 42.6 C: 39.5	ARCO	I/II
Ang 2015 [67]	Hebei	T: 45 (28/17) C: 44 (30/14)	T: 43.6 ± 7.4 C: 44.7 ± 6.8	Ficat	II/III
Feng 2014 [68]	Shandong	T: 60 (NA) C:60 (NA)	T: 35.3 C: 35.3	ARCO	II/III
Zhang 2014 [69]	Jiangsu	T: 52 (41/11) C: 51 (42/9)	T: 45.8 ± 6.3 C: 44.9 ± 5.9	ARCO	I/II
Cheng 2014 [70]	Jiangsu	T: 15 (9/6) C: 15 (8/7)	T: 37.11 ± 5.61 C: 38.00 ± 5.71	Ficat	I/II
Zhao 2012 [71]	Shandong	T: 25 (17/8) C: 23 (16/7)	T: 36.3 C: 35.2	ARCO	I/II
Lu 2012 [72]	Hunan	T: 12 (8/4) C: 12 (9/3)	T: 37.8 ± 11.7 C: 38.6 ± 12.3	Ficat	I/II
Su 2012 [73]	Fujian	T: 18 (15/3) C: 18 (14/4)	T: 37.91 ± 12.14 C: 38.12 ± 11.08	Ficat	I/II
Du 2011 [74]	Henan	T: 45 (39/6) C: 45 (28/7)	T: 36 C: 42	Ficat	I/II

Note: *ARCO* Association Research Circulation Osseous; *C* control group; *NA* not available; *T* treatment group

**Table 2:** Intervention characteristics of included studies

Inclusion Studies	Treatment group	Control group	Duration (month)	Follow-up (month)	Outcome
Zheng 2022 [28]	Oral Xianling Gubao capsule (3 capsules, bid) + C	CD + BG	12	12	HHS Occurrence of adverse reaction
Zhang 2022 [29]	Oral Duzhongjiangu granule (12 g, tid) + C	ESWT (2–3 sessions/1 week)	3	3	HHS
Shen 2022 [30]	Oral Taoren decoction (300 mL, bid) + C	Oral Alendronate sodium (70 mg, qw)	3	NA	HHS VAS Occurrence of adverse reaction
Sun 2022 [31]	Oral Guguton Huaisiyu Capsule (5 capsules, tid) + C	CD + BG	6	NA	HHS VAS Imaging improvement
Li 2022 [32]	Oral Sanqi Huogu Pill (6 g, bid) + C	ESWT (1 session/2 days)	1	NA	HHS VAS
Han 2021 [33]	Oral Xianling Gubao Capsule (1.5 g, bid) + C	ESWT (1 session/2 days)	3	NA	HHS VAS Occurrence of adverse reaction
Liao 2021 [34]	Oral Huangu Bone Healing Compound (25 mL, q2d) + C	ESWT (1 session/2 days)	3	3	HHS Imaging improvement
Han 2021 [35]	Oral Gubi tongxiao granule (5 g, tid) + C	CD + BG	6	12	HHS VAS
Liao 2021 [36]	Oral Fuyang Revitalizing Bone Pill (1 dose, bid) + C	ESWT (1 session/2 days)	3	3	HHS Imaging improvement
Sun 2021 [37]	Oral Self-made prescription (1 dose, bid) + C	CD + BG	3	NA	HHS
Liu 2020 [38]	Oral Self-made prescription (300 mL, bid) + C	ESWT (20 min/1 session, 1 session/1 day)	3	NA	HHS
Du 2020 [39]	Oral Tiansui capsule (6 capsules, tid) + C	ESWT (25 min/1 session, 2 sessions/1 week)	3	12	HHS VAS Imaging improvement
Zhao 2020 [40]	Oral Bushen huogu capsule (5 capsules, tid) + C	ESWT (2 sessions/1 week)	3	NA	HHS Occurrence of adverse reaction
Yan 2020 [41]	Oral Supplemented No.1 Zhuli Decoction (300 mL, bid) + C	CD + BG	3	6	HHS Imaging improvement Occurrence of adverse reaction
Wei 2019 [42]	Oral Bushen Huoxue Decoction (1 dose, NA) + C	Osteotomy	6	12	HHS VAS
Sun 2019 [43]	Oral Huoxue Shengu Decoction (1 dose, bid) + C	CD + BG	6	24	HHS Imaging improvement
Zhou 2019 [44]	Oral Sanjiao paste (10 mL, bid) + C	ESWT (1 session/1 week)	2	NA	HHS VAS
Zhan 2019 [45]	Oral Self-made Bu Gu Decoction (2 doses, bid) + C	Oral Alendronate sodium (70 mg, qw)	6	NA	VAS
Zhao 2019 [46]	Oral Guningwan (5 g, tid) + C	CD + BG	6	6	HHS VAS Imaging improvement
Cao 2018 [47]	Oral Modified Shentong Zhuyutang(1 dose, bid) + C	CD + BG	9	9	HHS Imaging improvement
Wang 2018 [48]	Oral Wenyang Bushen Decoction (1 dose, bid) + C	CD + BG	3	3	HHS
Song 2018 [49]	Oral Gugutou Huaisiyu Capsule (6 capsules, tid) + C	ESWT (2 sessions/1 week)	3	24	HHS VAS
Du 2018 [50]	Oral Shenqi Decoction (400 mL, bid) + C	Oral Alendronate sodium (10 mg)	3	6	HHS Occurrence of adverse reaction
Yuan 2018 [51]	Oral Yuanshi ShengmaiChenggu Tablet (3 tablets, tid) + C	ESWT (5 sessions/1 week)	6	12	HHS VAS
Liu 2017 [52]	Oral Shengu II Decoction (200 mL, bid) + C	CD + BG	2	NA	HHS
Lu 2017 [53]	Oral Syndrome differentiation (300 mL, bid) + C	Oral Alendronate sodium (70 mg, qw)	3	NA	HHS Occurrence of adverse reaction
Jiang 2017 [54]	Oral Gugutou Huaisiy Capsule (5 capsules, bid) + C	Porous tantalum rod	6	12	HHS Imaging improvement Occurrence of adverse reaction
Li 2017 [55]	Oral Busui Huoxue Jiangu Decoction (1 dose, bid) + C	CD + BG	3	NA	HHS VAS Imaging improvement
He 2017 [56]	Oral Shengu Decoction (200 mL, bid) + C	CD + BG	12	12	HHS Imaging improvement
Xu 2017 [57]	Oral Bushen Huoxue Decoction (1 dose, bid) + C	CD + BG	3	3	HHS Imaging improvement
Zhang 2016 [58]	Oral Bushen Huoxue Decoction (1 dose, NA) + C	CD + BG	6	12	HHS
Zhang 2016 [59]	Oral Tongluo Shenggu Decoction (1 dose, bid) + C	CD + BG	3	24	HHS VAS
Nong 2016 [60]	Oral Huoxue Jiangu Decoction (1 dose, bid) + C	CD + BG	3	12	HHS Imaging improvement
Tian 2016 [61]	Oral Gugutou Huaisiyu Capsule (5 capsules, tid) + C	Porous tantalum rod	6	NA	HHS Occurrence of adverse reaction
Zhu 2015 [62]	Oral Self-made prescription (200 mL, bid) + C	CD + BG	1	6	HHS VAS Imaging improvement
Liu 2015 [63]	Oral Huogu Decoction (1 dose, bid) + C	CD + BG	6	NA	HHS Imaging improvement
Zhou 2015 [64]	Oral Jiawei Qing E Pill (1 dose, bid) + C	Oral Western medicine (600 mg, qd)	6	6	HHS VAS
Wang 2015 [65]	Oral Jianbu Huqian Pill (1 dose, bid) + C	CD + BG	4	12	HHS Imaging improvement
Li 2015 [66]	Oral Lugui Shenggu Pill (10 g, bid) + C	CD	12	12	HHS Imaging improvement
Ang 2015 [67]	Oral Busui Huoxue Jiangu Decoction (300 mL, bid) + C	CD + BG	3	12	HHS Imaging improvement
Feng 2014 [68]	Oral Xianling Gubao Capsule (3 capsules, bid) + C	CD + BG	12	NA	HHS
Zhang 2014 [69]	Oral Huoxue Busui Decoction (150 mL, tid) + C	CD + BG	3	3	HHS Imaging improvement
Cheng 2014 [70]	Oral Syndrome differentiation (1 dose, bid) + C	CD + BG	3	12	HHS Imaging improvement
Zhao 2012 [71]	Oral Bushen Huoxue Decoction (1 dose, bid) + C	Porous tantalum rod	3	13	HHS
Lu 2012 [72]	Oral Bushen Huoxue Decoction (300 mL, bid) + C	CD + BG	1	NA	HHS Imaging improvement
Su 2012 [73]	Oral Syndrome differentiation (1 dose, bid) + C	CD + BG	12	NA	Imaging improvement
Du 2011 [74]	Oral Syndrome differentiation (1 dose, bid) + C	CD + BG	3	12	HHS Imaging improvement

*BG* bone-grafting; *C* control group; *CD* core decompression; *ESWT* extracorporeal shock wave; *HHS* Harris hip score therapy; *NA* not available; *T* treatment group; *VAS* visual analogue scale

### Assessment of the risk of bias

**Fig. 2:**
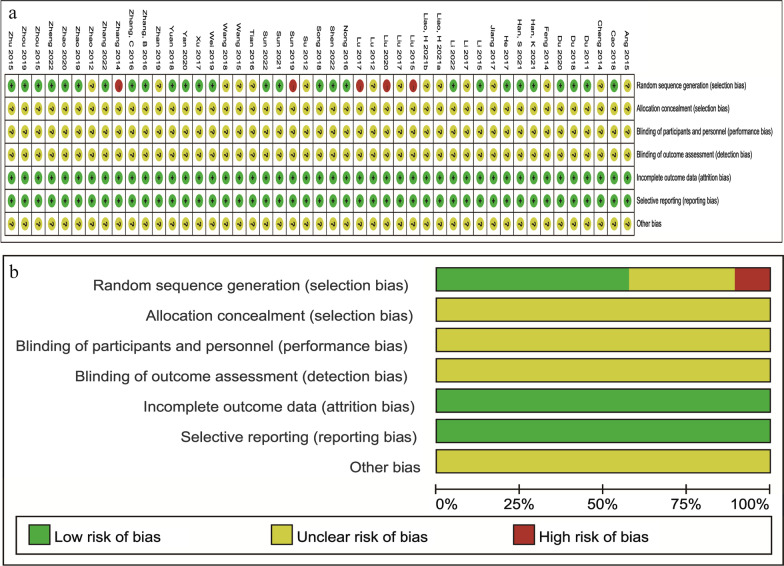
Risk of bias graph in the included studies. **a** Risk of bias summary **b** Risk of bias graph

The Cochrane risk of bias tool was used to systematically evaluate the quality of the 47 RCTs [28–74]. Twenty-seven studies [28–33, 35, 37, 39–42, 44, 46, 47, 49–51, 56–60, 62, 64, 66, 74] clearly described correct random sequence generation methods (computerized random method or random number method, etc.), so they were rated as having a low risk of bias for that domain. Fifteen studies [34, 36, 45, 48, 52, 54, 55, 61, 65, 67, 68, 70–73] only referred to randomized groups but did not specify the specific method, so they were rated as having an unclear risk of bias for this domain. Five studies [38, 43, 53, 63, 69] did not mention randomization (admission order grouping or wishes, etc.), so they were rated as having a high risk of bias for this domain. Since allocation procedures and binding methods were not mentioned in any of the included RCTs, they were all rated as having an unclear risk of bias for this domain. The integrity of outcome data and selective reporting of all researchers were judged to be at low risk of bias, as no data deficiencies and specified indicators were completely reported. However, no details were found in all studies for other biases, and thus, they were rated as having an unclear risk of bias (Fig. 2).

### Meta-analysis results

#### Harris score

**Fig. 3:**
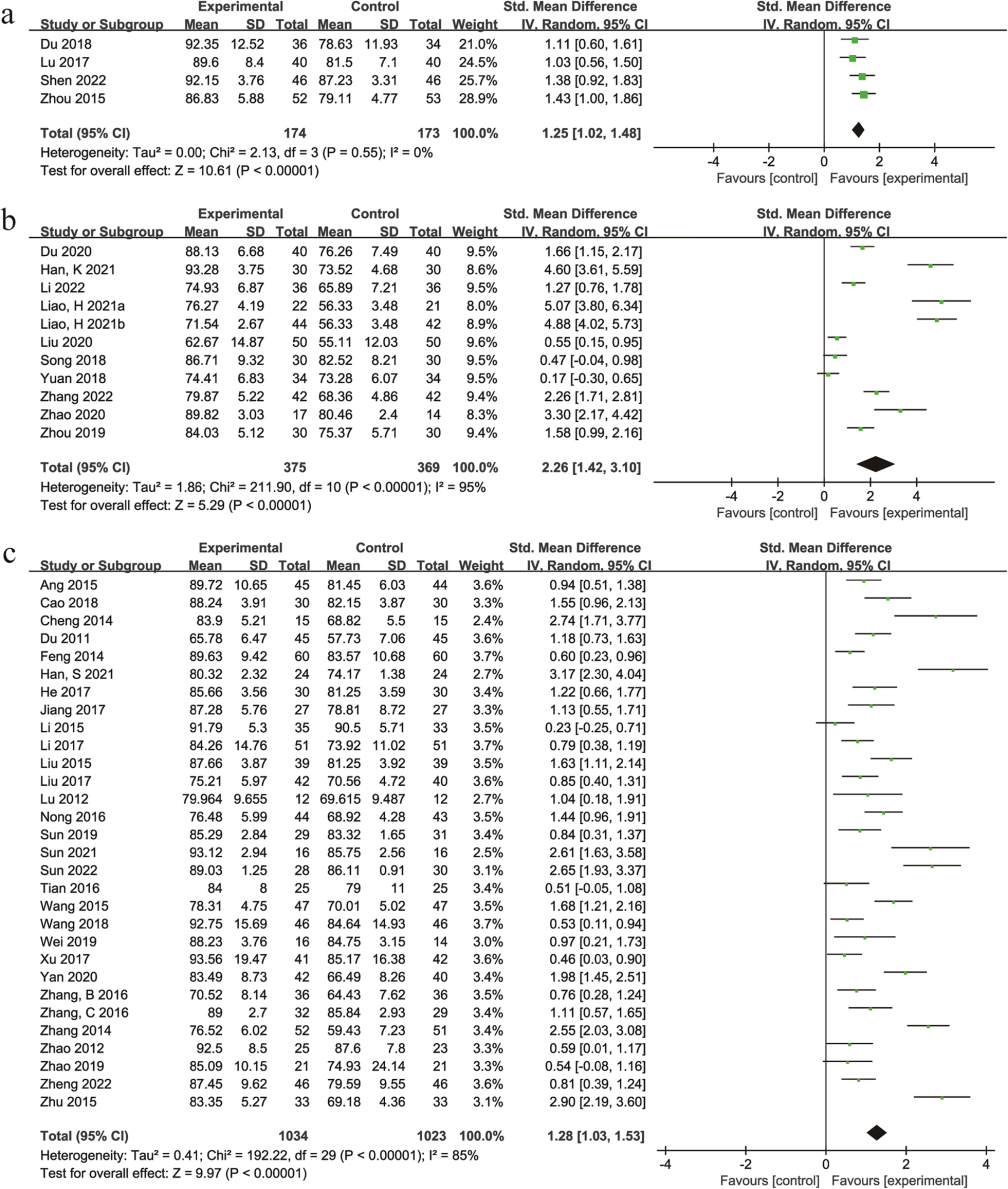
Forest plot of the meta-analysis of the Harris score: **a** traditional Chinese medicine + Western medicine versus Western medicine **b** traditional Chinese medicine + physiotherapy versus physiotherapy **c** traditional Chinese medicine + hip preservation surgery versus hip preservation surgery

A total of 45 studies [28–44, 46–72, 74] with 3148 participants compared the Harris score between the treatment groups and control groups. Three studies [32, 62, 72] had a treatment duration of 1 month, 2 studies [44, 52] had a treatment duration of 2 months, 23 studies [29, 30, 33, 34, 36–41, 48–50, 53, 55, 57, 59, 60, 67, 69–71, 74] had a treatment duration of 3 months, 1 study [65] had a treatment duration of 4 months, 11 studies [31, 35, 42, 43, 46, 51, 54, 58, 61, 63, 64] had a treatment duration of 6 months, 1 study [47] had a treatment duration of 9 months, and 4 studies [28, 56, 66, 68] had a treatment duration of 12 months. Due to differences in the durations of treatment and herbal compositions, a more rigorous random effects model was used. The results showed that the TCM + Western medicine versus Western medicine groups [30, 50, 53, 64] (SMD = 1.25, 95% Cl: 1.02 to 1.48, *P* < 0.00001) (Fig. 3a). The TCM + physiotherapy versus physiotherapy group [29, 32–34, 36, 38–40, 44, 49, 51] (SMD = 2.26, 95% Cl: 1.42 to 3.10, *P* < 0.00001) (Fig. 3b). The TCM + hip preservation surgery versus hip preservation surgery group [28, 31, 35, 37, 41–43, 46–48, 52, 54–63, 65–72, 74] (SMD = 1.28, 95% Cl: 1.03 to 1.53, *P* < 0.00001) (Fig. 3c). The results indicating that TCM combined with Western medicine was superior to Western medicine alone in improving the Harris score.

### Visual analogue scale score

**Fig. 4:**
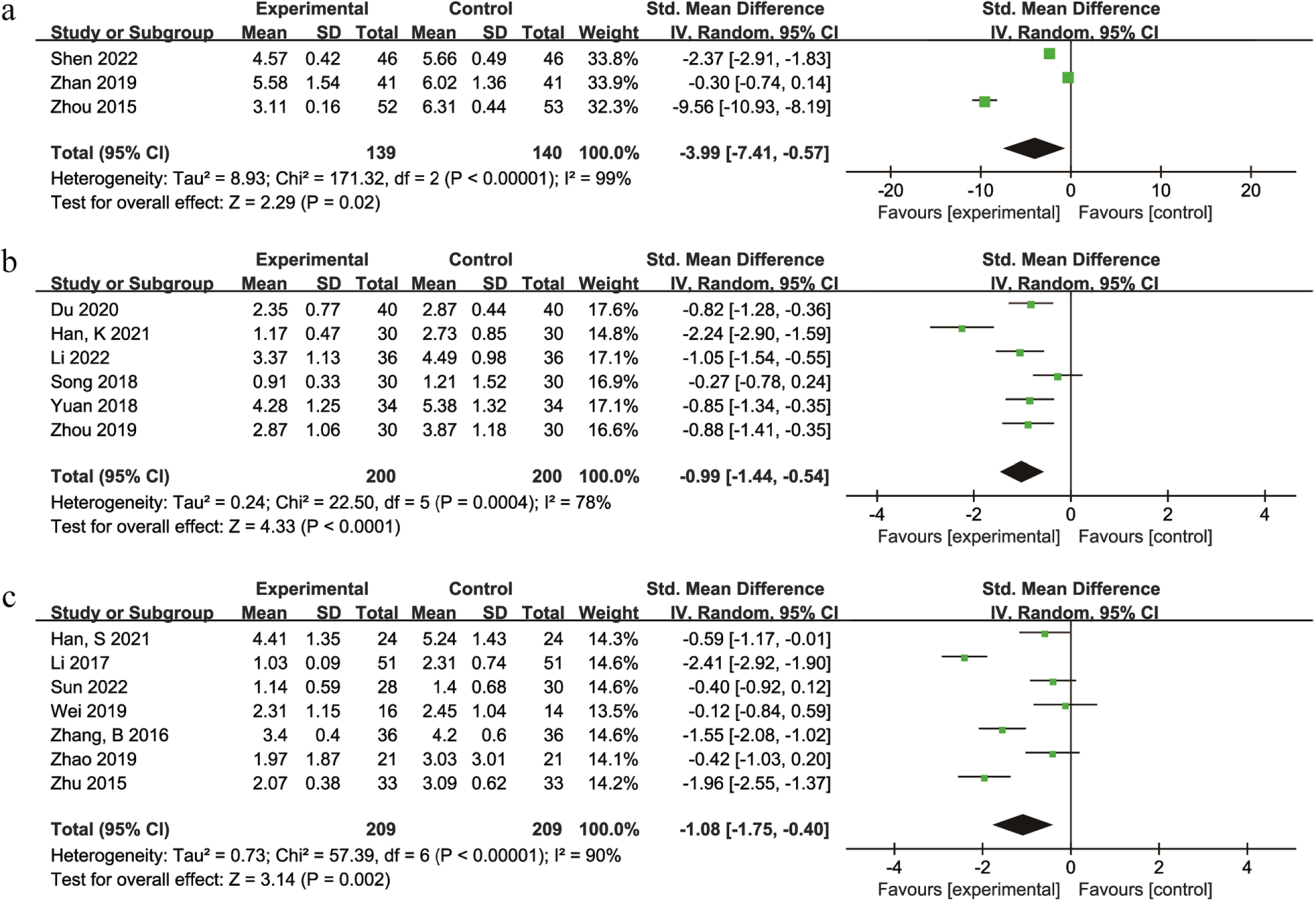
Forest plot of the meta-analysis of the visual analogue scale score: **a** traditional Chinese medicine + Western medicine versus Western medicine **b** traditional Chinese medicine + physiotherapy versus physiotherapy **c** traditional Chinese medicine + hip preservation surgery versus hip preservation surgery

A total of 16 studies [30–33, 35, 39, 42, 44–46, 49, 51, 55, 59, 62, 64] with 1097 participants compared the visual analogue scale score between the treatment groups and control groups. Two studies [32, 62] had a treatment duration of 1 month, 1 study [44] had a treatment duration of 2 months, 6 studies [30, 33, 39, 49, 55, 59] had a treatment duration of 3 months, and 7 studies [31, 35, 42, 45, 46, 51, 64] had a treatment duration of 6 months. Due to differences in the durations of treatment and herbal compositions, a more rigorous random effects model was used. The results showed that the TCM + Western medicine versus Western medicine groups [30, 45, 64] (SMD = −3.99, 95% Cl: −7.41 to −0.57, *P* = 0.02) (Fig. 4a). The TCM + physiotherapy versus physiotherapy group [32, 33, 39, 44, 49, 51] (SMD = −0.99, 95% Cl: −1.44 to −0.54, *P* < 0.0001) (Fig. 4b). The TCM + hip preservation surgery versus hip preservation surgery group [31, 35, 42, 46, 55, 59, 62] (SMD = −1.08, 95% Cl: −1.75 to −0.40, *P* = 0.002) (Fig. 4c). The results indicating that TCM combined with Western medicine was superior to Western medicine alone in relieving joint pain.

### Imaging improvement

**Fig. 5:**
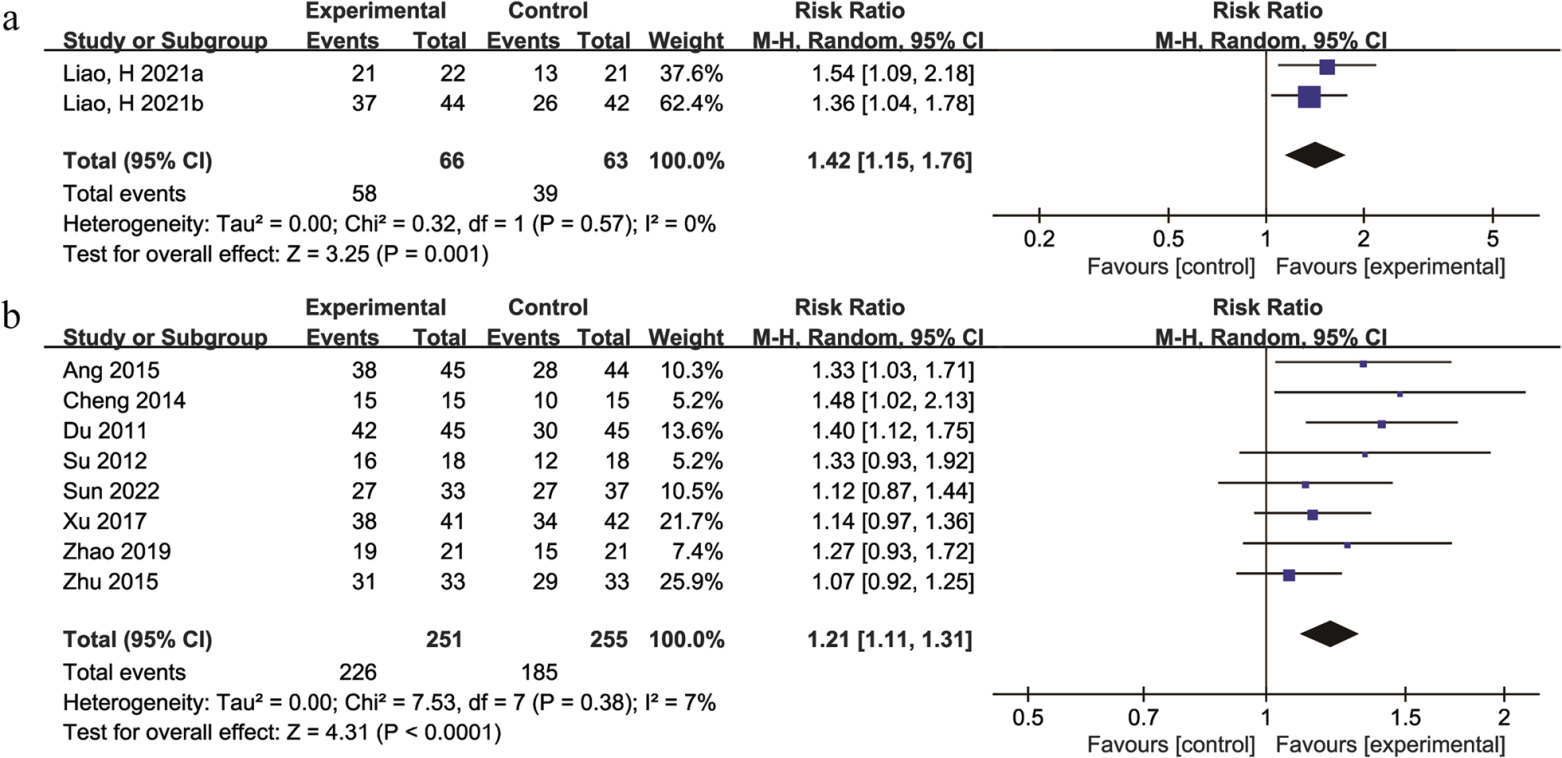
Forest plot of the meta-analysis of the imaging improvement: **a** traditional Chinese medicine + physiotherapy versus physiotherapy **b** traditional Chinese medicine + hip preservation surgery versus hip preservation surgery

A total of 23 studies [31, 34, 36, 39, 41, 43, 46, 47, 54–57, 60, 62, 63, 65–67, 69, 70, 72–74] with 1579 participants compared improvements in imaging results between the treatment groups and control groups. Ten studies described the number of cases that showed improvement, 1 study [65] described changes in ONFH volume, 3 studies [41, 55, 72] reported scores on the hip imaging scale, 5 studies [43, 54, 60, 66, 69] evaluated the collapse of the femoral head, and 4 studies [39, 47, 56, 63] examined the Association Research Circulation Osseous or Ficat stage criteria. A total of 10 studies [31, 34, 36, 46, 57, 62, 67, 70, 73, 74] were included for analysis, of which 1 study [62] had a treatment duration of 1 month, 7 studies [34, 36, 57, 67, 70, 74] had a treatment duration of 3 months, 2 studies [31, 46] had a treatment duration of 6 months, and 1 study [73] had a treatment duration of 12 months. Due to differences in the durations of treatment and herbal compositions, a more rigorous random effects model was used. The results showed that the TCM + physiotherapy versus physiotherapy group [34, 36] (RR = 1.42, 95% Cl: 1.15 to 1.76, *P* = 0.001) (Fig. 5a). The TCM + hip preservation surgery versus hip preservation surgery group [31, 46, 57, 62, 67, 70, 73, 74] (RR = 1.21, 95% Cl: 1.11 to 1.31, *P* < 0.0001) (Fig. 5b). The comprehensive analysis showed that both the number of cases that showed improvements and the description of the change in volume and score of femoral head necrosis indicated that TCM combined with Western medicine was superior to Western medicine alone in terms of imaging improvement.

### Occurrence of adverse reaction

**Fig. 6:**
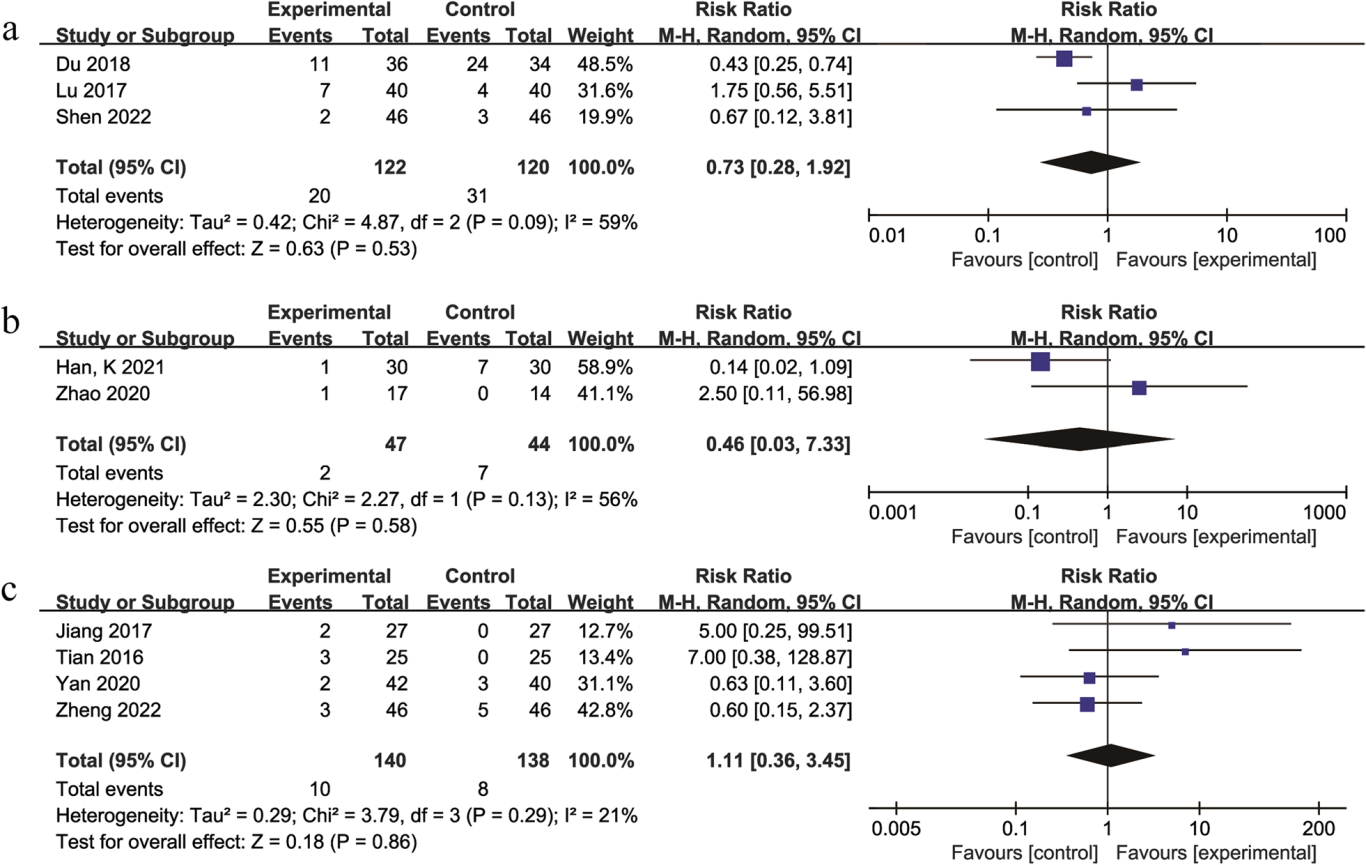
Forest plot of the meta-analysis of the occurrence of adverse reaction: **a** traditional Chinese medicine + Western medicine versus Western medicine **b** traditional Chinese medicine + physiotherapy versus physiotherapy **c** traditional Chinese medicine + hip preservation surgery versus hip preservation surgery

A total of 9 studies [28, 30, 33, 40, 41, 50, 53, 54, 61] with 611 participants reported the occurrence of adverse reaction, including abdominal pain, nausea, and constipation, and no life-threatening events were reported in the treatment group or control group (Additional file 1: Table S3). Six studies [30, 33, 40, 41, 50, 53] had a treatment duration of 3 months, 2 studies [54, 61] had a treatment duration of 6 months, and 1 study [28] had a treatment duration of 12 months. Due to differences in the durations of treatment and herbal compositions, a more rigorous random effects model was used. The results showed that the TCM + Western medicine versus Western medicine groups [30, 50, 53] (RR = 0.73, 95% Cl: 0.28 to 1.92, *P* = 0.53) (Fig. 6a). The TCM + physiotherapy versus physiotherapy group [33, 40] (RR = 0.46, 95% Cl: 0.03 to 7.33, *P* = 0.58) (Fig. 6b). The TCM + hip preservation surgery versus hip preservation surgery group [28, 41, 54, 61] (RR = 1.11, 95% Cl: 0.36 to 3.45, *P* = 0.86) (Fig. 6c). It is suggested that TCM combined with Western medicine does not increase the incidence of adverse effects.

### Sensitivity analysis

To determine the effect of each individual clinical trial on the pooled effect size, we excluded each study from the analysis. In the test for the overall effect *P*-value of visual analogue scale score in the TCM + Western medicine versus Western medicine group, there were changes in the outputs after excluding each study. After removing the study conducted by Shen’s study [30], Zhan’s study [45], and Zhou’s study [64], the result showed no significance. Results also showed that in Du’s study [50], and Lu’s study [53], the I^2^ of occurrence of adverse reaction in the TCM + Western medicine versus Western medicine group dropped to 0%. The I^2^ of occurrence of adverse reaction in the TCM + hip preservation surgery versus hip preservation surgery group decreased to 0% after the elimination of study [61]. After removing any study from other outcome indicators, no other important changes were found, reflecting that the results of the meta-analysis were robust. The details of sensitivity analysis are presented in Additional file 1: Table S4-S7.

### Publication bias

The funnel plotting for the Harris score in the TCM + physiotherapy versus physiotherapy group and TCM + hip preservation surgery versus hip preservation surgery group showed asymmetry, thus indicating potential publication bias (Additional file 1: Fig. S1-S2). Therefore, Egger’s test was used to analyze the publication bias. The results of Egger’s test showed possible publication bias in the analysis results of the Harris score in the TCM + physiotherapy versus physiotherapy group and TCM + hip preservation surgery versus hip preservation surgery group (*P* < 0.05) (Additional file 1: Table S8). This bias may be related to the fact that articles with negative results are not easily published, the small sample size of some studies, and the fact that the literature included in this study was all in Chinese, so there was language bias. We did not perform a publication bias test for the other observational indicators. Due to the small number of studies (< 10).

## Discussion

In patients with ONFH, the effectiveness of TCM combined with Western medicine remains controversial [75]. Previous systematic reviews [76, 77] reported that compared with controls, TCM treatment had a consistent treatment effect in patients. Recently, TCM has attracted considerable attention, and considerable research has been conducted on the effects of oral traditional Chinese herbal medicine on ONFH. Consequently, this meta-analysis of studies updates the literature and further evaluates the impact of TCM combined with Western medicine on patients with ONFH. A total of 47 articles with a total of 3266 patients were included in this meta-analysis. The overall posttreatment Harris score, visual analogue scale score, and imaging improvement were better in the TCM combined with the Western medicine group than in the Western medicine group. There was no significant difference between the two groups in the occurrence of adverse reaction. Additionally, funnel plots and Egger’s test were constructed for the Harris score in the TCM + physiotherapy versus physiotherapy group and TCM + hip preservation surgery versus hip preservation surgery group included in the study and indicated potential publication bias.

The Harris score has been widely used to evaluate the efficacy of hip preservation therapy by comprehensively evaluating pain, hip function, and daily activities. The results showed that TCM combined with Western medicine is positively effective in improving joint function. According to a study by Zheng’s study [28], hip preservation surgery combined with TCM therapy resulted in a significant increase in the Harris score after 12 months, which aligns with findings from relevant literature [35], further confirming the clinical efficacy of the combination of Chinese and Western medicine therapy. Similarly, du’s study [39] observes higher Harris score in the treatment group. On the one hand, physical therapy can loosen the tissues around the hip joint and remodel the necrotic femoral head’s bone structure [78, 79]. On the other hand, TCM can enhance the blood supply to the femoral head and promote the repair of osteonecrosis [80]. These complementary effects mutually improve joint function.

We identified the visual analogue scale score as the primary outcome. The results demonstrated that TCM combined with Western medicine contributes to a reduction in pain intensity. Han’s study [35] showed a significant decrease in visual analogue scale score for 24 subjects who received postoperative oral Chinese medicine treatment. Core decompression by reducing pressure within the femoral head and increasing blood flow to the necrotic area. Additionally, oral Chinese medicine promotes local blood flow and inhibits platelet agglutination, further mitigating hip pain in patients [46]. Zheng’s study [81] suggested that TCM may achieve pain relief by decreasing levels of serum TNF-α and CRP.

On the contrary, our study found the combination of TCM and Western medicine can delay femoral head collapse. Yan’s study [41] discovered that TCM may effectively delay femoral head collapse by improving intraosseous microcirculation, inhibiting osteoclast proliferation, and promoting bone tissue regeneration. Similarly, animal experiments by Zhou’s study [18] demonstrated that TCM may reduce the occurrence of empty bone lacunae and stimulate bone formation by modulating the Wnt/β-catenin signaling pathway.

Our review has produced consistent findings with another two reviews [76, 77] published in the English language on TCM combined with Western medicine for ONFH. The primary outcome from one of their reviews used total effective rate to estimate the efficacy [77]. However, the total effective rate to ONFH was rarely used in the RCTs. The series of scales, such as the Harris score scale and visual analogue scale are widely used worldwide. Hence, symptom severity measured by Harris score scale and pain relief measured by visual analogue scale are necessary to be assessed. In this review, we also focused on the use of imaging improvement to assess efficacy. Compared to the previous systematic reviews [76, 77], our review provides a variety of new perspectives. First, more rigorous inclusion and exclusion criteria could increase the quality of evidence and reduce the risk of bias. Additionally, we indicated the most commonly used traditional Chinese herbal medicines in our analysis and listed them in Additional file 1: Table S1. There are three main sources of heterogeneity: (1) differences in measures of the same outcome indicators between studies; (2) the period of the treatment course was inconsistent across the included studies; (3) different levels of experience and competence among clinicians. The safety of oral traditional Chinese herbal medicine is a key concern of the article due to the specific nature of traditional Chinese herbal medicine. Only 9 RCTs in this study reported adverse effects, including abdominal pain, nausea, and constipation. The reasons for this may include the following: (1) the researchers did not consider observing safety when developing the protocol; or (2) the investigators guided the medication promptly to detect adverse reactions and actively intervened in the treatment; thus, they did not cause serious adverse reactions.

Nevertheless, this meta-analysis has many shortcomings. (1) Only 27 of the included studies mentioned scientific allocation, and most studies did not report allocation concealment, blinding, and other circumstances with the potential risk of measurement bias and implementation bias. (2) Since TCM adopts holistic dialectical thinking, it is necessary to add or subtract medication to address individual patient differences, making it difficult to measure the impact of drug addiction or subtraction on efficacy, and confounding factors are difficult to control, thus increasing heterogeneity. (3) Most studies did not follow up with patients, and there is a lack of data on the long-term effects of TCM on improving function and relieving pain. (4) There was inconsistency between the protocol and the manuscript, which can lead to bias. Thus, it should be gradually improved and enhanced in future studies.

ONFH belongs to the category of bone erosion in Chinese medicine, and its cause is mostly due to deficiency of the liver and kidney, lack of qi and blood, resulting in the development of phlegm, and stagnation of blood vessels. The herbs Danshen (Radix Salviae Miltiorrhizae), Niuxi (Radix Achyranthis Bidentatae), and Huangqi (Radix Astragali) are commonly used in clinical treatment and have the effect of tonifying the liver, benefiting the kidneys and activating blood circulation to remove blood stasis [82, 83]. Modern research has shown that Radix Salviae Miltiorrhizae has mainly anti-inflammatory, immunomodulatory, and glucolipid metabolic effects, which can effectively improve the hematopoietic function of patients [84]. The active ingredients of Radix Achyranthis Bidentatae can achieve osteoprotective effects by inhibiting adipogenesis, the inflammatory response, and osteoblast apoptosis in a series of ways [85]. Astragalus polysaccharide has the ability to promote the proliferation and differentiation of osteoblasts and inhibit inflammatory factors [86].

A number of common problems in the included articles were identified in this study. First, for any intervention, safety evaluation is as important as efficacy evaluation. In particular, the current clinical studies of TCM for the treatment of ONFH are dominated by self-prepared formulas, the adverse effects of which are less clear than those of listed proprietary Chinese medicines, and a standardized safety evaluation would help to improve acceptance [41, 50]. Second, the implementation of blinding is difficult due to the specificity of herbal therapy, but it is still recommended that this step be implemented where possible; if not, then it should be detailed in the limitations of the study section. Finally, in clinical trials of TCM for ONFH, the diagnostic criteria for TCM evidence should be clarified to help better exploit the value of the study and guide practice [39, 47, 73].

## Conclusion

TCM combined with Western medicine may be a superior treatment approach for ONFH compared with the use of Western medicine alone. However, the included studies in this meta-analysis had a low overall level of evidence. Therefore, large-scale and high-quality RCTs are needed for further evaluation to provide a rational and effective treatment plan for the clinical management of early- and mid-stage ONFH.

### Supplementary Information

**Additional file 1.** Search strategies. **Table S1.** Composition of TCM in the study. **Table S2.** Characteristics of physiotherapy. **Table S3.** Details of reported adverse events from included studies. **Table S4.** Seneitivity analysis for Harris score. **Table S5.** Seneitivity analysis for visual analogue scale score. **Table S6.** Seneitivity analysis for imaging improvement. **Table S7.** Seneitivity analysis for occurence of adverse reaction. **Fig. S1.** Funnel plot for publication bias of the literature reporting the Harris score: traditional Chinese medicine + physiotherapy vs. physiotherapy. **Fig. S2.** Funnel plot for publication bias of the literature reporting the Harris score: traditional Chinese medicine + hip preservation surgery vs. hip preservation surgery. **Table S8.** Publication bias of the included studies.

## Abbreviations

CI: Confidence interval
ONFH: Osteonecrosis of the femoral head
RCTs: Randomized controlled trials
RR: Risk ratio
SMD: Standardized mean difference
TCM: Traditional Chinese medicine
