# Supplementary material for: Efficacy and safety of traditional Chinese medicine in the treatment of osteonecrosis of the femoral head
Source: J Orthop Surg Res. 2023 Aug 14;18:600. doi: 10.1186/s13018-023-04086-9 (PMC10424453; doi:10.1186/s13018-023-04086-9)
Supplement: Supplementary file 1 — Additional file 1. Search strategies. Table S1. Composition of TCM in the study. Table S2. Characteristics of physiotherapy. Table S3. Details of reported adverse events from included studies. Table S4. Seneitivity analysis for Harris score. Table S5. Seneitivity analysis for visual analogue scale score. Table S6. Seneitivity analysis for imaging improvement. Table S7. Seneitivity analysis for occurence of adverse reaction. Fig. S1. Funnel plot for publication bias of the literature reporting the Harris score: traditional Chinese medicine + physiotherapy vs. physiotherapy. Fig. S2. Funnel plot for publication bias of the literature reporting the Harris score: traditional Chinese medicine + hip preservation surgery vs. hip preservation surgery. Table S8. Publication bias of the included studies. [file 13018_2023_4086_MOESM1_ESM.docx]

**Supplementary Material**

Supplement to: Efficacy and safety of Traditional Chinese Medicine in the treatment of osteonecrosis of the femoral head

Contents

Search strategies 2

Table S1. Composition of TCM in the study. 4

Table S2. Characteristics of physiotherapy. 11

Table S3. Details of reported adverse events from included studies. 12

Table S4. Seneitivity analysis for Harris score. 13

Table S5. Seneitivity analysis for visual analogue scale score. 15

Table S6. Seneitivity analysis for imaging improvement. 16

Table S7. Seneitivity analysis for occurence of adverse reaction. 17

Fig. S1. Funnel plot for publication bias of the literature reporting the Harris score: traditional Chinese medicine + physiotherapy vs. physiotherapy. 18

Fig. S2. Funnel plot for publication bias of the literature reporting the Harris score: traditional Chinese medicine + hip preservation surgery vs. hip preservation surgery. 19

Table S8. Publication bias of the included studies. 20

**Search strategies**

*PubMed:*

#1 ((((traditional Chinese medicine [Title/Abstract]) OR (Chinese medicinal herbs [Title/Abstract]) OR (pill [Title/Abstract])) OR (decoction [Title/Abstract])) OR (capsule [Title/Abstract])

#2 ("osteonecrosis of the femoral head" [Mesh]) OR (((((((femur head necrosis [Title/Abstract])) OR (aseptic necrosis of femur head [Title/Abstract])) OR (avascular necrosis of femur head [Title/Abstract])) OR (avascular necrosis of femoral head [Title/Abstract])) OR (ischemic necrosis of femoral head [Title/Abstract])) OR (ONFH [Title/Abstract])) OR (FHN [Title/Abstract])) OR (ANFH [Title/Abstract])

#3 #1 AND #2

*Web of Science:*

#1 TS=(traditional Chinese medicine OR Chinese medicinal herbs OR pill OR decoction OR capsule)

#2 TS=(osteonecrosis of the femoral head OR femur head necrosis OR aseptic necrosis of femur head OR avascular necrosis of femur head OR avascular necrosis of femoral head OR ischemic necrosis of femoral head OR ONFH OR FHN OR ANFH)

#3 #1 AND #2

*Embase:*

#1 'traditional Chinese medicine'/exp

#2 'Chinese medicinal herbs':ab,ti OR 'pill':ab,ti OR 'decoction':ab,ti OR 'capsule':ab,ti

#3 #1 OR #2

#4 'osteonecrosis of the femoral head'/exp

#5 'femur head necrosis':ab,ti OR 'aseptic necrosis of femur head':ab,ti OR 'avascular necrosis of femur head':ab,ti OR 'ischemic necrosis of femoral head':ab,ti OR 'ONFH':ab,ti OR 'FHN':ab,ti OR 'ANFH':ab,ti

#6 #4 OR #5

#7 #3 AND #6

*Cochrane Central Register of Controlled Trials：*

#1 (traditional Chinese medicine):ti,ab,kw OR (Chinese medicinal herbs):ti,ab,kw OR (pill):ti,ab,kw OR (decoction):ti,ab,kw OR (capsule):ti,ab,kw

#2 MeSH descriptor: [osteonecrosis of the femoral head] explode all trees

#3 (femur head necrosis):ti,ab,kw OR (aseptic necrosis of femur head):ti,ab,kw OR (avascular necrosis of femur head):ti,ab,kw OR (ischemic necrosis of femoral head):ti,ab,kw OR (ONFH):ti,ab,kw OR (FHN):ti,ab,kw OR (ANFH):ti,ab,kw

#4 #2 OR #3

#5 #1 AND #4

*Chinese National Knowledge Infrastructure (CNKI)：*
( ( ( ( (主题=中医 或者 题名=中医 或者 v_subject=中英文扩展(中医) 或者 title=中英文扩展(中医)) 或者 (主题=中药 或者 题名=中药 或者 v_subject=中英文扩展(中药) 或者 title=中英文扩展(中药)) ) 或者 ( (主题=中草药 或者 题名=中草药 或者 v_subject=中英文扩展(中草药) 或者 title=中英文扩展(中草药)) 或者 (主题=丸 或者 题名=丸 或者 v_subject=中英文扩展(丸) 或者 title=中英文扩展(丸)) ) ) 或者 ( (主题=方 或者 题名=方 或者 v_subject=中英文扩展(方) 或者 title=中英文扩展(方)) 或者 (主题=汤药 或者 题名=汤药 或者 v_subject=中英文扩展(汤药) 或者 title=中英文扩展(汤药)) ) ) 或者 (主题=胶囊 或者 题名=胶囊 或者 v_subject=中英文扩展(胶囊) 或者 title=中英文扩展(胶囊)) ) 并且 ( (主题=股骨头坏死 或者 题名=股骨头坏死 或者 v_subject=中英文扩展(股骨头坏死) 或者 title=中英文扩展(股骨头坏死)) 或者 (主题=股骨头缺血性坏死 或者 题名=股骨头缺血性坏死 或者 v_subject=中英文扩展(股骨头缺血性坏死) 或者 title=中英文扩展(股骨头缺血性坏死)) )

*Chinese Science and Technology Periodical database：*

(题名或关键词=中医 OR 题名或关键词=中药) OR 题名或关键词=中草药) OR 题名或关键词=丸) OR 题名或关键词=方) OR 题名或关键词=汤药) OR 题名或关键词=胶囊) AND (题名或关键词=股骨头坏死 OR 题名或关键词=股骨头缺血性坏死))

*WanFang database：*

((主题:(中医) or 主题:(中药) or 主题:(中草药) or 主题:(丸) or 主题:(方) or 主题:(汤药) or 主题:(胶囊)) and (主题:(股骨头坏死) or 主题:(股骨头缺血性坏死)

*Chinese Biological Medicine database：*

#1 "中医"[不加权:扩展]

#2 ("中药"[常用字段:智能] OR "中草药"[常用字段:智能] OR "丸"[常用字段:智能] OR "方"[常用字段:智能] OR "汤药"[常用字段:智能] OR "胶囊"[常用字段:智能])

#3 ("股骨头坏死"[常用字段:智能] OR "股骨头缺血性坏死"[常用字段:智能])

#4 ((#1) OR (#2))

#5 ((#3) AND ((#4)

**Table S1**

Composition of TCM in the study.

| Inclusion  studies | Prescription | Medicine |
| --- | --- | --- |
| Zheng  2022 | Xianling Gubao capsule | Yinyanghuo (Epimedium Herb), Xuduan (Radix Dipsaci), Buguzhi (Fructus Psoraleae), Shengdihuang (Radix Rehmanniae), Zhimu (Rhizoma Anemarrhenae), Danshen (Radix Salviae Miltiorrhizae) |
| Zhang  2022 | Duzhongjiangu granule | Duzhong (Eucommia Bark), Xuduan (Radix Dipsaci), Jixueteng (Suberect Spatholobus Stem Caulis Spatholobi), Danggui (Radix Angelicae Sinensis), Gouqi (Barbary Wolfberry Fruit Fructus Lycil), Niuxi (Radix Achyranthis Bidentatae), Sanqi (Radix Notoginseng), Weilingxian (Clematis Root) |
| Shen  2022 | Taoren decoction | Taoren (Semen Persicae) 15g, Honghua (Flos Carthami) 10g, Huangqi (Radix Astragali) 10g, Dangshen (Medicinal Changium Root Radix Changii) 10g, Danggui (Radix Angelicae Sinensis) 12g, Shudihuang (Radix Rehmanniae Preparata) 24g, Chuanxiong (Rhizoma Ligustici Chuanxiong) 15g, Chishao (Radix Paeoniae Rubra) 15g, Duzhong (Eucommia Bark) 30g, Xuduan (Radix Dipsaci) 30g, Niuxi (Radix Achyranthis Bidentatae) 30g, Chuanwu (Commom Monkshood Mother Root Radix Aconiti  ) 9g, Caowu (Kusnezoff Monkshood Root Radix Aconiti Kusnezoffii) 6g, Gancao (Radix Glycyrrhizae) 6g |
| Sun  2022 | Guguton Huaisiyu capsule | Duzhong (Eucommia Bark), Xuduan (Radix Dipsaci), Buguzhi (Fructus Psoraleae), Huangqi (Radix Astragali), Danggui (Radix Angelicae Sinensis), Danshen (Radix Salviae Miltiorrhizae), Jixueteng (Suberect Spatholobus Stem Caulis Spatholobi), Tubiechong (Ground Beeltle Eupolyphaga seu Steleophaga), Xuanshen (Figwort Root Radix Scrophulariae), Lianqiao (Fructus Forsythiae Suspensae), Shuizhi (Hirudo), Ruxiang (Frankincense), Moyao (Myrrh), Xuejie (Dragons Blood Resina Draconis), Fuling (Poria), Guizhi (Ramulus Cinnamomi) |
| Li  2022 | Sanqi Huogu pill | Sanqi (Radix Notoginseng), Gusuibu (Fortunes Drynaria Rhizome), Zirantong (Pyrite), Tubiechong (Ground Beeltle Eupolyphaga seu Steleophaga), Muxiang (Radix Aucklandiae), Yanhusuo (Rhizoma Corydalis), Taoren (Semen Persicae), Baishao (Radix paeoniae Alba), Xuduan (Radix Dipsaci), Liushenqu (Medicated Leaven Massa Medicata Fermentata), Niuxi (Radix Achyranthis Bidentatae), Sumu (Sappan Wood Ligum Sappan), Guijia (Tortoise Shell), Biejia (Turtle Shell) |
| Han  2021 | Xianling Gubao capsule | Yinyanghuo (Epimedium Herb), Xuduan (Radix Dipsaci), Zhimu (Rhizoma Anemarrhenae), Buguzhi (Fructus Psoraleae), Shudihuang (Radix Rehmanniae Preparata), Danshen (Radix Salviae Miltiorrhizae) |
| Liao  2021 | Hengu Bone Healing compound | Sanqi (Radix Notoginseng), Honghua (Flos Carthami) |
| Han  2021 | Gubi tongxiao granule | Danshen (Radix Salviae Miltiorrhizae) 15g, Buguzhi (Fructus Psoraleae) 15g, Chishao (Radix Paeoniae Rubra) 12g, Yinyanghuo (Epimedium Herb) 12g, Heshouwu (Radix Polygoni Multiflori) 12g, Chuanxiong (Rhizoma Ligustici Chuanxiong) 10g, Danggui (Radix Angelicae Sinensis) 10g, Xuduan (Radix Dipsaci) 10g, Tubiechong (Ground Beeltle Eupolyphaga seu Steleophaga) 10g, Rougui (Cortex Cinnamomi) 4g, Shuizhi (Hirudo) 3g, Gancao (Radix Glycyrrhizae) 6g |
| Liao  2021 | Fuyang Revitalizing Bone pill | Maqianzi (Nux Vomica Semen Strychni) 0.6g, Sanqi (Radix Notoginseng) 15g, Honghua (Flos Carthami) 20g, Ruxiang (Frankincense) 15g, Moyao (Myrrh) 15g, Shudihuang (Radix Rehmanniae Preparata) 30g, Rougui (Cortex Cinnamomi) 15g, Lujiaojiao (Antler gum) 6g, Cancao (Radix Glycyrrhizae) 9g |
| Sun  2021 | Self-made prescription | Gusuibu (Fortunes Drynaria Rhizome) 15g, Jianghuang (Turmeric Rhizoma Curcumae Longae) 10g, Yinyanghuo (Epimedium Herb) 15g, Duzhong (Eucommia Bark) 15g, Renshen (Radix Ginseng) 6g, Chenpi (Pericarpium Citri Reticulatae) 10g, Sanqi (Radix Notoginseng) 5g, Shuizhi (Hirudo) 3g, Tubiechong (Ground Beeltle Eupolyphaga seu Steleophaga) 10g |
| Liu  2020 | Self-made prescription | Huangqi (Radix Astragali) 30g, Danshen (Radix Salviae Miltiorrhizae) 15g, Shudihuang (Radix Rehmanniae Preparata)15g, Duzhong (Eucommia Bark) 10g, Shanyao (Rhizoma Dioscoreae) 15g, Niuxi (Radix Achyranthis Bidentatae) 6g, Gouqi (Barbary Wolfberry Fruit Fructus Lycil) 15g, Baishao (Radix paeoniae Alba) 10g, Gusuibu (Fortunes Drynaria Rhizome) 10g, Danggui (Radix Angelicae Sinensis) 15g, Dilong (Lumbricus) 10g |
| Du  2020 | Tiansui capsule | Gusuibu (Fortunes Drynaria Rhizome), Tubiechong (Ground Beeltle Eupolyphaga seu Steleophaga), Taoren (Semen Persicae), Honghua (Flos Carthami), Danshen (Radix Salviae Miltiorrhizae), Sanqi (Radix Notoginseng), Ruxiang (Frankincense), Moyao (Myrrh), Xuejie (Dragons Blood Resina Draconis), Dilong (Lumbricus) |
| Zhao  2020 | Bushen huogu capsule | Duzhong (Eucommia Bark) 10g, Xuduan (Radix Dipsaci) 10g, Gusuibu (Fortunes Drynaria Rhizome) 10g, Yinyanghuo (Epimedium Herb) 10g, Danshen (Radix Salviae Miltiorrhizae) 15g, Honghua (Flos Carthami) 10g, Chuanxiong (Rhizoma Ligustici Chuanxiong) 10g, Danggui (Radix Angelicae Sinensis) 10g, Shenjincao (Common Clubmoss Herb Herba Lycopodii) 15g, Lurong (Hairy Antler), Gancao (Radix Glycyrrhizae) |
| Yan  2020 | Supplemented No.1 Zhuli decoction | Fuling (Poria) 20g, Ganjiang (Dried Ginger Rhizoma Zingiberis) 20g, Qinjiao (Radix Gentianae Macrophyllae) 20g, Duhuo (Radix Angelicae Pubescentis) 20g, Fangji (Fangchi Root) 20g, Fangfeng (Radix Saposhnikovia Root) 20g, Shenma (Largetrifoliolious Bugbane Rhizome Rhizome Cimicifugae) 20g, Gegen (Radix Puerariae) 15g, Huangqi (Radix Astragali) 15g, Mahuang (Ephedra Herb) 15g, Rougui (Cortex Cinnamomi) 15g, Fuzi (Aconiti Lateralis radix Praeparata stem) 15g, Dilong (Lumbricus) 10g, Xinren (Bitter Apricot Seed Semen Armeniacae Amarum) 10g, Xixin (Herba Asari) 10g, Gancao (Radix Glycyrrhizae) 10g |
| Wei  2019 | Bushen Huoxue decoction | Duhuo (Radix Angelicae Pubescentis) 10g, Sangjisheng (Chinese Taxillus Twig Herba Taxilli) 10g, Yinyanghuo (Epimedium Herb) 10g, Buguzhi (Fructus Psoraleae) 10g, Mugua (Fructus Chaenomelis) 10g, Danggui (Radix Angelicae Sinensis) 10g, Danshen (Radix Salviae Miltiorrhizae) 12g, Shudihuang (Radix Rehmanniae Preparata) 10g, Chuanxiong (Rhizoma Ligustici Chuanxiong) 10g, Niuxi (Radix Achyranthis Bidentatae) 12g, Ruxiang (Frankincense) 10g, Moyao (Myrrh) 10g, Baizhu (Rhizoma Atractylodis Macrocephalae) 10g, Gancao (Radix Glycyrrhizae) 10g |
| Sun  2019 | Huoxue Shengu decoction | Danggui (Radix Angelicae Sinensis) 20g, Chuanxiong (Rhizoma Ligustici Chuanxiong) 20g, Chishao (Radix Paeoniae Rubra) 15g, Baishao (Radix paeoniae Alba) 15g, Shudihuang (Radix Rehmanniae Preparata) 12g, Honghua (Flos Carthami) 12g, Niuxi (Radix Achyranthis Bidentatae) 15g, Chuanwu (Commom Monkshood Mother Root Radix Aconiti) 6g, Tusizi (Semen Cuscutae) 15g, Dangshen (Medicinal Changium Root Radix Changii) 15g, Huangqi (Radix Astragali) 30g, Xuduan (Radix Dipsaci) 15g, Yanhusuo (Rhizoma Corydalis) 15g, Wugong (Centipede) 5g, Lujiaojiao (Antler gum) 10g, Maiya (Fructus hordei germinatus) 12g, Shanzha (Fructus Crataegi) 12g, Shenqu (Medicated Leaven Massa Medicata Fermentata) 12g |
| Zhou  2019 | Sanjiao paste | Ejiao (Colla Corii Asini) 90g, Guijiajiao (Tortoise Shell) 90g, Lujiaojiao (Antler gum) 90g, Chaihu (Radix Bupleuri) 90g, Yujin (Radix curcumae) 90g, Chuanlianzi (Melaleuca alternifolia) 90g, Shengdihuang (Radix Rehmanniae) 60g, Chishao (Radix Paeoniae Rubra) 60g, Tusizi (Semen Cuscutae) 60g, Hanliancao (Drynaria) 60g, Niuxi (Radix Achyranthis Bidentatae) 60g, Baizhi (Radix Angelicae Dahuricae) 60g, Baijiangcang (Stiff Siikworm) 60g, Baixianpi (Cortex Dictamni) 90g, Sanqi (Radix Notoginseng), Biejia (Turtle Shell) 60g, Danpi (Cortex Moutan Radicis) 60g, Yuzhu (Fragrant Solomonseal Rhizome) 60g, Maidong (Radix Ophiopogonis) 60g, Chuanshanjia (Pangolin Scales) 40g, Gegen (Radix Puerariae) 150g |
| Zhan  2019 | Self-made Bu Gu decoction | Xuejie (Dragons Blood Resina Draconis), Dilong (Lumbricus), Chuanxiong (Rhizoma Ligustici Chuanxiong), Dangshen (Medicinal Changium Root Radix Changii), Sangjisheng (Chinese Taxillus Twig Herba Taxilli), Gusuibu (Fortunes Drynaria Rhizome), Niuxi (Radix Achyranthis Bidentatae), Honghua (Flos Carthami), Xuduan (Radix Dipsaci), Ruxiang (Frankincense), Moyao (Myrrh), Gancao (Radix Glycyrrhizae) |
| Zhao  2019 | Guningwan | Weilingxian (Clematis Root), Wushaoshe (Razorback), Zhenzhu (Pearl), Tougucao (Bonesetter), Gouji (East Asian Tree Fern Rhizome), Danggui (Radix Angelicae Sinensis), Hupo (Amber Succinum), Chuanxiong (Rhizoma Ligustici Chuanxiong), Huangqi (Radix Astragali), Lurong (Hairy Antler) |
| Cao  2018 | Modified Shentong Zhuyutang | Xiangfu (Rhizoma Cyperi) 15g, Gusuibu (Fortunes Drynaria Rhizome) 20g, Chaihu (Radix Bupleuri) 15g, Chuanxiong (Rhizoma Ligustici Chuanxiong) 15g, Taoren (Semen Persicae) 15g, Honghua (Flos Carthami) 15g, Wulingzhi (Trogoterus Dung Faeces Trogopterori) 12g, Moyao (Myrrh) 12g, Danggui (Radix Angelicae Sinensis) 15g, Dilong (Lumbricus) 12g, Niuxi (Radix Achyranthis Bidentatae) 15g, Gancao (Radix Glycyrrhizae) 6g |
| Wang  2018 | Wenyang Bushen decoction | Huangqi (Radix Astragali) 15g, Bajitian (Medicinal Changium Root Radix Changii) 15g, Niuxi (Radix Achyranthis Bidentatae) 9g, Yujin (Radix curcumae) 9g, Danshen (Radix Salviae Miltiorrhizae) 9g, Gusuibu (Fortunes Drynaria Rhizome) 9g, Yinyanghuo (Epimedium Herb) 9g, Lujiaojiao (Antler gum) 6g, Gancao (Radix Glycyrrhizae) 3g, Sanqi (Radix Notoginseng) 3g |
| Song  2018 | Gugutou Huaisiyu capsule | Lurong (Hairy Antler) 2g, Duzhong (Eucommia Bark) 10g, Xuduan (Radix Dipsaci) 10g, Huangqi (Radix Astragali) 15g, Lianqiao (Fructus Forsythiae Suspensae) 10g, Danshen (Radix Salviae Miltiorrhizae) 20g, Jixueteng (Suberect Spatholobus Stem Caulis Spatholobi) 15g, Shuizhi (Hirudo) 5g, Ruxiang (Frankincense) 10g, Moyao (Myrrh) 10g |
| Du  2018 | Shenqi Decoction | Gusuibu (Fortunes Drynaria Rhizome) 20g, Shanyao (Rhizoma Dioscoreae) 20g, Shengdihuang (Radix Rehmanniae) 20g, Fuzi (Aconiti Lateralis radix Praeparata stem) 15g, Rougui (Cortex Cinnamomi) 15g, Niuxi (Radix Achyranthis Bidentatae) 15g, Duhuo (Radix Angelicae Pubescentis) 10g, Shanzhuyu (Fructus Corni) 10g, Danpi (Cortex Moutan Radicis) 10g |
| Yuan  2018 | Yuanshi ShengmaiChenggu tablet | Danshen (Radix Salviae Miltiorrhizae), Chuanxiong (Rhizoma Ligustici Chuanxiong), Jixueteng (Suberect Spatholobus Stem Caulis Spatholobi), Huangqi (Radix Astragali) |
| Liu  2017 | Shengu Ⅱ Decoction | Buguzhi (Fructus Psoraleae), Gusuibu (Fortunes Drynaria Rhizome), Duzhong (Eucommia Bark), Chuanxiong (Rhizoma Ligustici Chuanxiong), Honghua (Flos Carthami), Danshen (Radix Salviae Miltiorrhizae), Rougui (Cortex Cinnamomi), Jixueteng (Suberect Spatholobus Stem Caulis Spatholobi), Chuanshanjia (Pangolin Scales), Tubiechong (Ground Beeltle Eupolyphaga seu Steleophaga) |
| Lu  2017 | Syndrome differentiation | Taoren (Semen Persicae) 15g, Honghua (Flos Carthami) 10g, Danggui (Radix Angelicae Sinensis) 10g, Shengdihuang (Radix Rehmanniae) 10g, Niuxi (Radix Achyranthis Bidentatae) 10g, Chishao (Radix Paeoniae Rubra) 6g, Zhiqiao (Fructus Aurantii) 6g, Gancao (Radix Glycyrrhizae) 6g, Chuanxiong (Rhizoma Ligustici Chuanxiong) 5g OR Duhuo (Radix Angelicae Pubescentis) 10g, Sangjisheng (Chinese Taxillus Twig Herba Taxilli) 8g, Duzhong (Eucommia Bark) 8g, Niuxi (Radix Achyranthis Bidentatae) 8g, Xixin (Herba Asari) 8g, Qinjiao (Radix Gentianae Macrophyllae) 8g, Fuling (Poria) 8g, Rougui (Cortex Cinnamomi) 8g, Fangfeng (Radix Saposhnikovia Root) 8g, Chuanxiong (Rhizoma Ligustici Chuanxiong) 6g, Renshen (Radix Ginseng) 6g, Gancao (Radix Glycyrrhizae) 6g, Danggui (Radix Angelicae Sinensis) 6g, Baishao (Radix paeoniae Alba) 6g, Shengdihuang (Radix Rehmanniae) 6g |
| Jiang  2017 | Gugutou Huaisiy capsule | Lianqiao (Fructus Forsythiae Suspensae) 10g, Huangqi (Radix Astragali) 15g, Lurong (Hairy Antler) 2g, Jixueteng (Suberect Spatholobus Stem Caulis Spatholobi) 15g, Duzhong (Eucommia Bark) 10g, Danshen (Radix Salviae Miltiorrhizae) 20g, Shuizhi (Hirudo) 5g, Xuduan (Radix Dipsaci) 10g, Ruxiang (Frankincense) 10g, Moyao (Myrrh) 10g |
| Li  2017 | Busui Huoxue Jiangu decoction | Huangqi (Radix Astragali) 30g, Shudihuang (Radix Rehmanniae Preparata) 20g, Sanqi (Radix Notoginseng) 20g, Niuxi (Radix Achyranthis Bidentatae) 20g, Sumu (Sappan Wood Ligum Sappan) 15g, Honghua (Flos Carthami) 15g, Danshen (Radix Salviae Miltiorrhizae) 15g, Chuanxiong (Rhizoma Ligustici Chuanxiong) 15g, Baishao (Radix paeoniae Alba) 15g, Danggui (Radix Angelicae Sinensis) 15g, Gouqi (Barbary Wolfberry Fruit Fructus Lycil) 15g, Jixueteng (Suberect Spatholobus Stem Caulis Spatholobi) 15g, Shanyao (Rhizoma Dioscoreae) 15g, Fuling (Poria) 15g, Gusuibu (Fortunes Drynaria Rhizome) 15g |
| He  2017 | Shengu decoction | Luoshiteng (Chinese Starjasmine Stem Caulis Trachelospermi) 20g, Songlan (Pinellia) 15g, Duzhong (Eucommia Bark) 15g, Sifangteng (Tetragonal Vine) 10g, Shuitianqi (Waterfield Seven) 10g, Dayeqianjinba (Philippine Flemingia Root Radix Flemingiae Philippinensis) 10g, Diulebang (Throwing the Bat) 10g, Jixueteng (Suberect Spatholobus Stem Caulis Spatholobi) 20g |
| Xu  2017 | Bushen Huoxue decoction | Shudihuang (Radix Rehmanniae Preparata) 15g, Danshen (Radix Salviae Miltiorrhizae) 15g, Huangqi (Radix Astragali) 30g, Danggui (Radix Angelicae Sinensis) 15g, Chuanxiong (Rhizoma Ligustici Chuanxiong) 9g, Bajitian (Medicinal Changium Root Radix Changii) 15g, Dangshen (Medicinal Changium Root Radix Changii) 15g, Yinyanghuo (Epimedium Herb) 15g, Gouji (East Asian Tree Fern Rhizome) 15g, Buguzhi (Fructus Psoraleae) 15g, Sangjisheng (Chinese Taxillus Twig Herba Taxilli) 15g, Gusuibu (Fortunes Drynaria Rhizome) 15g, Gouteng (Ramulus Uncariae cum Uncis) 15g, Jixueteng (Suberect Spatholobus Stem Caulis Spatholobi) 15g, Baishao (Radix paeoniae Alba) 15g, Baizhu (Rhizoma Atractylodis Macrocephalae) 15g, Gegen (Radix Puerariae) 15g, Guizhi (Ramulus Cinnamomi) 9g, Chuanshanjia (Pangolin Scales) 9g, Quanxie (Scorpion) 9g, Wugong (Centipede) 5g, Dilong (Lumbricus) 9g, Baijiezi (Semen Sinapis Albae) 9g, Yanhusuo (Rhizoma Corydalis) 9g, Niuxi (Radix Achyranthis Bidentatae) 9g, Gancao (Radix Glycyrrhizae) 6g |
| Zhang  2016 | Bushen Huoxue decoction | Duhuo (Radix Angelicae Pubescentis) 10g, Sangjisheng (Chinese Taxillus Twig Herba Taxilli) 10g, Mugua (Fructus Chaenomelis) 10g, Yinyanghuo (Epimedium Herb) 10g, Buguzhi (Fructus Psoraleae) 10g, Danggui (Radix Angelicae Sinensis) 10g, Danshen (Radix Salviae Miltiorrhizae) 12g, Shudihuang (Radix Rehmanniae Preparata) 10g, Chuanxiong (Rhizoma Ligustici Chuanxiong) 10g, Niuxi (Radix Achyranthis Bidentatae) 12g, Moyao (Myrrh) 10g, Ruxiang (Frankincense) 10g, Baizhu (Rhizoma Atractylodis Macrocephalae) 12g, Gancao (Radix Glycyrrhizae) 6g |
| Zhang  2016 | Tongluo Shenggu decoction | Huangqi (Radix Astragali) 30g, Jixueteng (Suberect Spatholobus Stem Caulis Spatholobi) 30g, Lujiaojiao (Antler gum) 30g, Tubiechong (Ground Beeltle Eupolyphaga seu Steleophaga) 20g, Sanqi (Radix Notoginseng) 15g, Niuxi (Radix Achyranthis Bidentatae) 15g, Danshen (Radix Salviae Miltiorrhizae) 12g |
| Nong  2016 | Huoxue Jiangu decoction | Huangqi (Radix Astragali) 50g, Chuanxiong (Rhizoma Ligustici Chuanxiong) 10g, Danshen (Radix Salviae Miltiorrhizae) 10g, Sumu (Sappan Wood Ligum Sappan) 10g, Baishao (Radix paeoniae Alba) 20g, Fuling (Poria) 20g, Danggui (Radix Angelicae Sinensis) 15g, Sanqi (Radix Notoginseng) 15g, Niuxi (Radix Achyranthis Bidentatae) 15g |
| Tian  2016 | Gugutou Huaisiy capsule | Lurong (Hairy Antler) 2g, Duzhong (Eucommia Bark) 10g, Xuduan (Radix Dipsaci) 10g, Huangqi (Radix Astragali) 15g, Lianqiao (Fructus Forsythiae Suspensae) 10g, Danshen (Radix Salviae Miltiorrhizae) 20g, Jixueteng (Suberect Spatholobus Stem Caulis Spatholobi) 15g, Shuizhi (Hirudo) 5g, Ruxiang (Frankincense) 10g, Moyao (Myrrh) 10g |
| Zhu  2015 | Self-made prescription | Danggui (Radix Angelicae Sinensis) 10g, Chuanxiong (Rhizoma Ligustici Chuanxiong) 8g, Sanleng (Trigonometry) 8g, Chishao (Radix Paeoniae Rubra) 12g, Honghua (Flos Carthami) 12g, Taoren (Semen Persicae) 10g, Buguzhi (Fructus Psoraleae) 20g, Shudihuang (Radix Rehmanniae Preparata) 10g, Danshen (Radix Salviae Miltiorrhizae) 15g, Gancao (Radix Glycyrrhizae) 6g, Gusuibu (Fortunes Drynaria Rhizome) 10g, Tubiechong (Ground Beeltle Eupolyphaga seu Steleophaga) 8g, Huangqi (Radix Astragali) 10g, Ruxiang (Frankincense) 20g, Jixueteng (Suberect Spatholobus Stem Caulis Spatholobi) 20g, Yanhusuo (Rhizoma Corydalis) 15g, Shouwu (Radix Polygoni Multiflori) 15g |
| Liu  2015 | Huogu decoction | Gancao (Radix Glycyrrhizae) 3g, Honghua (Flos Carthami) 6g, Chishao (Radix Paeoniae Rubra) 6g, Chuanshanjia (Pangolin Scales) 6g, Dilong (Lumbricus) 6g, Danggui (Radix Angelicae Sinensis) 9g, Danshen (Radix Salviae Miltiorrhizae) 9g, Baishao (Radix paeoniae Alba) 9g, Xuduan (Radix Dipsaci) 12g, Taoren (Semen Persicae) 12g, Gusuibu (Fortunes Drynaria Rhizome) 12g, Niuxi (Radix Achyranthis Bidentatae) 15g |
| Zhou  2015 | Jiawei Qing E pill | Buguzhi (Fructus Psoraleae) , Taoren (Semen Persicae) , Roucongrong (Herba Cistanches) , Moyao (Myrrh) , Duzhong (Eucommia Bark) , Bajitian (Radix Morindae Officinalis) , Ruxiang (Frankincense) |
| Wang  2015 | Jianbu Huqian pill | Zhimu (Rhizoma Anemarrhenae) 10g, Huangbai (Amur Corktree Bark Cortex Phellodendri) 10g, Guibanjiao (Tortoise Shell) 10g, Lujiaojiao (Antler gum) 10g, Shudihuang (Radix Rehmanniae Preparata) 15g, Niuxi (Radix Achyranthis Bidentatae) 10g, Baishao (Radix paeoniae Alba) 10g, Duzhong (Eucommia Bark) 10g, Xuduan (Radix Dipsaci) 10g, Buguzhi (Fructus Psoraleae) 10g, Suoyang (Songaria Cynomorium Herb Herba Cynomorii) 10g, Tusizi (Semen Cuscutae) 10g, Danggui (Radix Angelicae Sinensis) 10g, Renshen (Radix Ginseng) 10g, Qianghuo (Incised Notopterygium Rhizome) 10g, Baizhu (Rhizoma Atractylodis Macrocephalae) 10g, Huangqi (Radix Astragali) 10g |
| Li  2015 | Lugui Shenggu pill | Lujiaojiao (Antler gum) 10g, Guiban (Tortoise Shell) 10g, Duzhong (Eucommia Bark) 10g, Shudihuang (Radix Rehmanniae Preparata) 10g, Danpi (Cortex Moutan Radicis) 10g |
| Ang  2015 | Busui Huoxue Jiangu decoction | Huangqi (Radix Astragali) 50g, Honghua (Flos Carthami) 10g, Danshen (Radix Salviae Miltiorrhizae) 10g, Chuanxiong (Rhizoma Ligustici Chuanxiong) 10g, Shudihuang (Radix Rehmanniae Preparata) 15g, Sanqi (Radix Notoginseng) 15g, Niuxi (Radix Achyranthis Bidentatae) 15g, Sumu (Sappan Wood Ligum Sappan) 15g, Baishao (Radix paeoniae Alba) 12g, Danggui (Radix Angelicae Sinensis) 12g, Gouqi (Barbary Wolfberry Fruit Fructus Lycil) 12g, Jixueteng (Suberect Spatholobus Stem Caulis Spatholobi) 30g, Shanyao (Rhizoma Dioscoreae) 30g, Fuling (Poria) 20g, Gusuibu (Fortunes Drynaria Rhizome) 30g |
| Feng  2014 | Xianling Gubao capsule | Yinyanghuo (Epimedium Herb), Xuduan (Radix Dipsaci), Buguzhi (Fructus Psoraleae), Shengdihuang (Radix Rehmanniae), Danshen (Radix Salviae Miltiorrhizae), Zhimu (Rhizoma Anemarrhenae) |
| Zhang  2014 | Huoxue Busui decoction | Danshen (Radix Salviae Miltiorrhizae) 30g, Huangqi (Radix Astragali) 30g, Danggui (Radix Angelicae Sinensis) 10g, Baishao (Radix paeoniae Alba) 10g, Yinyanghuo (Epimedium Herb) 10g, Quanxie (Scorpion) 10g, Baizhu (Rhizoma Atractylodis Macrocephalae) 9g, Fuling (Poria) 9g, Duzhong (Eucommia Bark) 9g, Niuxi (Radix Achyranthis Bidentatae) 15g, Gusuibu (Fortunes Drynaria Rhizome) 15g, Shuizhi (Hirudo) 6g, Rougui (Cortex Cinnamomi) 6g, Xianmao (Centaurium) 6g, Lurong (Hairy Antler) 2g, Wugong (Centipede) 5g, Gancao (Radix Glycyrrhizae) 6g |
| Cheng  2014 | Syndrome differentiation | Not available |
| Zhao  2012 | Bushen Huoxue decoction | Shudihuang (Radix Rehmanniae Preparata),Huangqi (Radix Astragali), Danggui (Radix Angelicae Sinensis), Danshen (Radix Salviae Miltiorrhizae), Honghua (Flos Carthami), Chuanxiong (Rhizoma Ligustici Chuanxiong), Gusuibu (Fortunes Drynaria Rhizome), Duzhong (Eucommia Bark), Xuduan (Radix Dipsaci), Niuxi (Radix Achyranthis Bidentatae), Bajitian (Medicinal Changium Root Radix Changii) |
| Lu  2012 | Bushen Huoxue decoction | Shudihuang (Radix Rehmanniae Preparata) 15g, Duzhong (Eucommia Bark) 15g, Gouji (East Asian Tree Fern Rhizome) 15g, Xuduan (Radix Dipsaci) 15g, Niuxi (Radix Achyranthis Bidentatae) 15g, Danggui (Radix Angelicae Sinensis) 20g, Chuanxiong (Rhizoma Ligustici Chuanxiong) 10g, Danshen (Radix Salviae Miltiorrhizae) 15g, Chishao (Radix Paeoniae Rubra) 20g, Sanqi (Radix Notoginseng) 10g, Gancao (Radix Glycyrrhizae) 10g |
| Su  2012 | Syndrome differentiation | Not available |
| Du  2011 | Syndrome differentiation | Qinjiao (Radix Gentianae Macrophyllae) 3g, Chuanxiong (Rhizoma Ligustici Chuanxiong) 6g, Taoren (Semen Persicae) 9g, Honghua (Flos Carthami) 9g, Gancao (Radix Glycyrrhizae) 6g, Qianghuo (Incised Notopterygium Rhizome) 3g, Moyao (Myrrh) 6g, Danggui (Radix Angelicae Sinensis) 9g, Wulingzhi (Trogoterus Dung Faeces Trogopterori) 6g, Xiangfu (Rhizoma Cyperi) 3g, Niuxi (Radix Achyranthis Bidentatae) 9g, Dilong (Lumbricus) 6g OR Duhuo (Radix Angelicae Pubescentis) 6g, Fangfeng (Radix Saposhnikovia Root) 6g, Niuxi (Radix Achyranthis Bidentatae) 6g, Sangjisheng (Chinese Taxillus Twig Herba Taxilli) 18g, Qinjiao (Radix Gentianae Macrophyllae) 12g, Duzhong (Eucommia Bark) 12g, Danggui (Radix Angelicae Sinensis) 12g, Fuling (Poria) 12g, Danshen (Radix Salviae Miltiorrhizae) 12g, Shudihuang (Radix Rehmanniae Preparata) 15g, Baishao (Radix paeoniae Alba) 10g, Xixin (Herba Asari) 3g, Gancao (Radix Glycyrrhizae) 3g, Rougui (Cortex Cinnamomi) 2g OR Fuling (Poria) 266g, Zhiqiao (Fructus Aurantii) 133g, Banxia (Rhizoma Pinelliae) 533g, Mangxiao (Mirabilitum) 66g, Shengjiang (Ginger) 133g OR Huangqi (Radix Astragali) 9g, Chishao (Radix Paeoniae Rubra) 9g, Guizhi (Ramulus Cinnamomi) 9g, Shengjiang (Ginger) 18g, Dazao (Chinese Dates) 10g OR Huangbai (Amur Corktree Bark Cortex Phellodendri) 240g, Guiban (Tortoise Shell) 120g, Zhimu (Rhizoma Anemarrhenae) 60g, Shudihuang (Radix Rehmanniae Preparata) 60g, Chenpi (Pericarpium Citri Reticulatae) 60g, Baishao (Radix paeoniae Alba) 60g, Suoyang (Songaria Cynomorium Herb Herba Cynomorii) 45g, Gougu (Dog bone) 30g, Ganjiang (Dried Ginger Rhizoma Zingiberis) 15g |

**Table S2**

Characteristics of physiotherapy.

| Inclusion  studies | Pulses | Energy flux density | Duration of treatment | Treatment course |
| --- | --- | --- | --- | --- |
| Zhang, 2022 | 600 | 0.18-0.25 mJ/mm^2^ | Not available | 2-3 sessions / 1 week |
| Li, 2022 | 1000 | Not available | Not available | 1 session / 2 days |
| Han, 2021 | 3000 | 0.16 mJ/mm^2^ | Not available | 1 session / 2 days |
| Liao, 2021 | 1000 | 0.20-0.35 mJ/mm^2^ | Not available | 1 session / 2 days |
| Liao, 2021 | 1000 | 0.20-0.35 mJ/mm^2^ | Not available | 1 session / 2 days |
| Liu, 2020 | NA | Not available | 20 min | 1 session / 1 day |
| Du, 2020 | 2500-3000 | Not available | 25 min | 2 sessions / 1 week |
| Zhao, 2020 | 1500 | Not available | Not available | 2 sessions / 1 week |
| Zhou, 2019 | NA | Not available | Not available | 1 session / 1 week |
| Song, 2018 | 3600 | 0.16 mJ/mm^2^ | Not available | 2 sessions / 1 week |
| Yuan, 2018 | 3000 | 0.14-0.16 mJ/mm^2^ | Not available | 5 sessions / 1 week |

**Table S3**

Details of reported adverse events from included studies.

| Inclusion  studies | Adverse event |
| --- | --- |
| Zheng  2022 [28] | T:3 (deep vein thrombosis=2, heterotopic ossification=1); C:5 (deep vein thrombosis=3, muscular atrophy=1, femoral nerve traction=1). |
| Shen  2022 [30] | T:2 (abdominal pain=1, constipation=1); C:3 (constipation=1, dyspepsia=1, headache=1). |
| Han  2021 [33] | T:1 (swelling and redness=1); C:7 (pain=1, deep vein thrombosis=4, swelling and redness=2). |
| Zhao  2020 [40] | T:1 (stomachache=1); C:0. |
| Yan  2020 [41] | T:2 (inflammatory hyperplasia=2); C:3 (inflammatory hyperplasia=2, adhesion=1). |
| Du  2018 [50] | T:11 (abdominal pain=3, constipation=1, nausea=2, dyspepsia=4, urinary red blood cell=1); C:24 (abdominal pain=8, constipation=4, nausea=3, dyspepsia=6, leukocyte elevation=1, urinary red blood cell=2). |
| Lu  2017 [53] | T:7 (insomnia=4, anxiety=3); C:4 (insomnia=4, anxiety=1). |
| Jiang  2017 [54] | T:2 (stomachache=2); C:0. |
| Tian  2016 [61] | T:3 (stomachache=3); C:0. |

**Table S4**

Seneitivity analysis for Harris score.

| Outcomes | Eliminated study | Heterogeneity | | SMD | 95% CI | P Value |
| --- | --- | --- | --- | --- | --- | --- |
|  |  | P Value | I^2^ (%) |  |  |  |
| TCM + Western medicine | None | 0.55 | 0 | 1.25 | 1.02 to 1.48 | <0.00001 |
|  | Shen 2022 [30] | 0.42 | 0 | 1.21 | 0.94 to 1.48 | <0.00001 |
|  | Du 2018 [50] | 0.42 | 0 | 1.29 | 1.03 to 1.55 | <0.00001 |
|  | Lu 2017 [53] | 0.61 | 0 | 1.32 | 1.06 to 1.59 | <0.00001 |
|  | Zhou 2015 [64] | 0.55 | 0 | 1.18 | 0.90 to 1.45 | <0.00001 |
| TCM + physiotherapy | None | <0.00001 | 95 | 2.26 | 1.42 to 3.10 | <0.00001 |
|  | Zhang 2022 [29] | <0.00001 | 96 | 2.27 | 1.35 to 3.18 | <0.00001 |
|  | Li 2022 [32] | <0.00001 | 96 | 2.37 | 1.43 to 3.32 | <0.00001 |
|  | Han 2021 [33] | <0.00001 | 95 | 2.03 | 1.22 to 2.83 | <0.00001 |
|  | Liao 2021 [34] | <0.00001 | 95 | 2.01 | 1.19 to 2.82 | <0.00001 |
|  | Liao 2021 [36] | <0.00001 | 94 | 1.98 | 1.22 to 2.73 | <0.00001 |
|  | Liu 2020 [38] | <0.00001 | 95 | 2.45 | 1.52 to 3.37 | <0.00001 |
|  | Du 2020 [39] | <0.00001 | 96 | 2.33 | 1.39 to 3.28 | <0.00001 |
|  | Zhao 2020 [40] | <0.00001 | 96 | 2.16 | 1.30 to 3.03 | <0.00001 |
|  | Zhou 2019 [44] | <0.00001 | 96 | 2.34 | 1.41 to 3.27 | <0.00001 |
|  | Song 2018 [49] | <0.00001 | 95 | 2.45 | 1.54 to 3.36 | <0.00001 |
|  | Yuan 2018 [51] | <0.00001 | 95 | 2.48 | 1.60 to 3.36 | <0.00001 |
| TCM + hip preservation surgery | None | <0.00001 | 85 | 1.25 | 1.03 to 1.53 | <0.00001 |
|  | Zheng 2022 [28] | <0.00001 | 85 | 1.30 | 1.04 to 1.56 | <0.00001 |
|  | Sun 2022 [31] | <0.00001 | 85 | 1.23 | 0.99 to 1.48 | <0.00001 |
|  | Han 2021 [35] | <0.00001 | 84 | 1.22 | 0.98 to 1.47 | <0.00001 |
|  | Sun 2021 [37] | <0.00001 | 85 | 1.24 | 0.99 to 1.49 | <0.00001 |
|  | Yan 2020 [41] | <0.00001 | 85 | 1.25 | 1.00 to 1.51 | <0.00001 |
|  | Wei 2019 [42] | <0.00001 | 85 | 1.29 | 1.03 to 1.55 | <0.00001 |
|  | Sun 2019 [43] | <0.00001 | 85 | 1.30 | 1.04 to 1.56 | <0.00001 |
|  | Zhao 2019 [46] | <0.00001 | 85 | 1.31 | 1.05 to 1.56 | <0.00001 |
|  | Cao 2018 [47] | <0.00001 | 85 | 1.27 | 1.01 to 1.53 | <0.00001 |
|  | Wang 2018 [48] | <0.00001 | 85 | 1.31 | 1.05 to 1.57 | <0.00001 |
|  | Liu 2017 [52] | <0.00001 | 85 | 1.30 | 1.04 to 1.56 | <0.00001 |
|  | Jiang 2017 [54] | <0.00001 | 85 | 1.29 | 1.03 to 1.55 | <0.00001 |
|  | Li 2017 [55] | <0.00001 | 85 | 1.30 | 1.04 to 1.56 | <0.00001 |
|  | He 2017 [56] | <0.00001 | 85 | 1.28 | 1.02 to 1.54 | <0.00001 |
|  | Xu 2017 [57] | <0.00001 | 85 | 1.31 | 1.05 to 1.57 | <0.00001 |
|  | Zhang 2016 [58] | <0.00001 | 85 | 1.29 | 1.03 to 1.55 | <0.00001 |
|  | Zhang 2016 [59] | <0.00001 | 85 | 1.30 | 1.04 to 1.56 | <0.00001 |
|  | Nong 2016 [60] | <0.00001 | 85 | 1.28 | 1.02 to 1.54 | <0.00001 |
|  | Tian 2016 [61] | <0.00001 | 85 | 1.31 | 1.05 to 1.56 | <0.00001 |
|  | Zhu 2015 [62] | <0.00001 | 83 | 1.22 | 0.98 to 1.46 | <0.00001 |
|  | Liu 2015 [63] | <0.00001 | 85 | 1.27 | 1.01 to 1.53 | <0.00001 |
|  | Wang 2015 [65] | <0.00001 | 85 | 1.27 | 1.01 to 1.52 | <0.00001 |
|  | Li 2015 [66] | <0.00001 | 84 | 1.32 | 1.06 to 1.57 | <0.00001 |
|  | Ang 2015 [67] | <0.00001 | 85 | 1.29 | 1.03 to 1.56 | <0.00001 |
|  | Feng 2014 [68] | <0.00001 | 85 | 1.31 | 1.05 to 1.57 | <0.00001 |
|  | Zhang 2014 [69] | <0.00001 | 83 | 1.23 | 0.99 to 1.47 | <0.00001 |
|  | Cheng 2014 [70] | <0.00001 | 85 | 1.24 | 0.99 to 1.49 | <0.00001 |
|  | Zhao 2012 [71] | <0.00001 | 85 | 1.30 | 1.05 to 1.56 | <0.00001 |
|  | Lu 2012 [72] | <0.00001 | 85 | 1.29 | 1.03 to 1.54 | <0.00001 |
|  | Du 2011 [74] | <0.00001 | 85 | 1.29 | 1.02 to 1.55 | <0.00001 |

**Table S5**

Seneitivity analysis for visual analogue scale score.

| Outcomes | Eliminated study | Heterogeneity | | SMD | 95% CI | P Value |
| --- | --- | --- | --- | --- | --- | --- |
|  |  | P Value | I^2^ (%) |  |  |  |
| TCM + Western medicine | None | <0.00001 | 99 | -3.99 | -7.41 to -0.57 | 0.02 |
|  | Shen 2022 [30] | <0.00001 | 99 | -4.91 | -13.98 to 4.17 | 0.29 |
|  | Zhan 2019 [45] | <0.00001 | 99 | -5.94 | -12.98 to 1.11 | 0.10 |
|  | Zhou 2015 [64] | <0.00001 | 97 | -1.33 | -3.36 to 0.70 | 0.20 |
| TCM + physiotherapy | None | 0.0004 | 78 | -0.99 | -1.44 to -0.54 | <0.0001 |
|  | Li 2022 [32] | 0.0002 | 82 | -0.99 | -1.54 to -0.43 | 0.0005 |
|  | Han 2021 [33] | 0.26 | 24 | -0.78 | -1.03 to -0.52 | <0.00001 |
|  | Du 2020 [39] | 0.0002 | 82 | -1.03 | -1.59 to -0.47 | 0.0003 |
|  | Zhou 2019 [44] | 0.0002 | 82 | -1.02 | -1.57 to -0.47 | 0.0003 |
|  | Song 2018 [49] | 0.005 | 73 | -1.13 | -1.58 to -0.68 | <0.00001 |
|  | Yuan 2018 [51] | 0.0002 | 82 | -1.03 | -1.58 to -0.47 | 0.0003 |
| TCM + hip preservation surgery | None | <0.00001 | 90 | -1.08 | -1.75 to -0.40 | 0.002 |
|  | Sun 2022 [31] | <0.00001 | 90 | -1.19 | -1.93 to -0.45 | 0.002 |
|  | Han 2021 [35] | <0.00001 | 91 | -1.16 | -1.92 to -0.39 | 0.003 |
|  | Wei 2019 [42] | <0.00001 | 90 | -1.22 | -1.93 to -0.52 | 0.0007 |
|  | Zhao 2019 [46] | <0.00001 | 90 | -1.18 | -1.93 to -0.44 | 0.002 |
|  | Li 2017 [55] | <0.0001 | 83 | -0.85 | -1.43 to -0.27 | 0.004 |
|  | Zhang 2016 [59] | <0.00001 | 91 | -0.99 | -1.78 to -0.21 | 0.01 |
|  | Zhu 2015 [62] | <0.00001 | 90 | -0.93 | -1.66 to -0.20 | 0.01 |

**Table S6**

Seneitivity analysis for imaging improvement.

| Outcomes | Eliminated study | Heterogeneity | | RR | 95% CI | P Value |
| --- | --- | --- | --- | --- | --- | --- |
|  |  | P Value | I^2^ (%) |  |  |  |
| TCM + physiotherapy | None | 0.57 | 0 | 1.42 | 1.15 to 1.76 | 0.001 |
|  | Liao 2021 [34] | - | - | 1.36 | 1.04 to 1.78 | 0.03 |
|  | Liao 2021 [36] | - | - | 1.54 | 1.09 to 2.18 | 0.01 |
| TCM + hip preservation surgery | None | 0.38 | 7 | 1.21 | 1.11 to 1.31 | <0.0001 |
|  | Sun 2022 [31] | 0.28 | 20 | 1.23 | 1.11 to 1.35 | <0.0001 |
|  | Zhao 2019 [46] | 0.28 | 19 | 1.21 | 1.10 to 1.33 | 0.0001 |
|  | Xu 2017 [57] | 0.31 | 16 | 1.23 | 1.11 to 1.36 | <0.0001 |
|  | Zhu 2015 [62] | 0.68 | 0 | 1.25 | 1.14 to 1.38 | <0.00001 |
|  | Ang 2015 [67] | 0.34 | 11 | 1.19 | 1.09 to 1.31 | 0.0002 |
|  | Cheng 2014 [70] | 0.40 | 3 | 1.19 | 1.09 to 1.29 | <0.0001 |
|  | Su 2012 [73] | 0.31 | 16 | 1.20 | 1.10 to 1.32 | <0.0001 |
|  | Du 2011 [74] | 0.51 | 0 | 1.17 | 1.08 to 1.28 | 0.0003 |

**Table S7**

Seneitivity analysis for occurence of adverse reaction.

| Outcomes | Eliminated study | Heterogeneity | | RR | 95% CI | P Value |
| --- | --- | --- | --- | --- | --- | --- |
|  |  | P Value | I^2^ (%) |  |  |  |
| TCM + Western medicine | None | 0.09 | 59 | 0.73 | 0.28 to 1.92 | 0.53 |
|  | Shen 2022 [30] | 0.03 | 79 | 0.79 | 0.20 to 3.16 | 0.74 |
|  | Du 2018 [50] | 0.36 | 0 | 1.31 | 0.50 to 3.41 | 0.58 |
|  | Lu 2017 [53] | 0.64 | 0 | 0.45 | 0.27 to 0.75 | 0.002 |
| TCM + physiotherapy | None | 0.13 | 56 | 0.46 | 0.03 to 7.33 | 0.58 |
|  | Han 2021 [33] | - | - | 2.50 | 0.11 to 56.98 | 0.57 |
|  | Zhao 2020 [40] | - | - | 0.14 | 0.02 to 1.09 | 0.06 |
| TCM + hip preservation surgery | None | 0.29 | 21 | 1.11 | 0.36 to 3.45 | 0.86 |
|  | Zheng 2022 [28] | 0.26 | 27 | 1.90 | 0.37 to 9.82 | 0.44 |
|  | Yan 2020 [41] | 0.18 | 41 | 1.80 | 0.31 to 10.31 | 0.51 |
|  | Jiang 2017 [54] | 0.29 | 20 | 0.88 | 0.27 to 2.83 | 0.83 |
|  | Tian 2016 [61] | 0.42 | 0 | 0.78 | 0.28 to 2.15 | 0.63 |


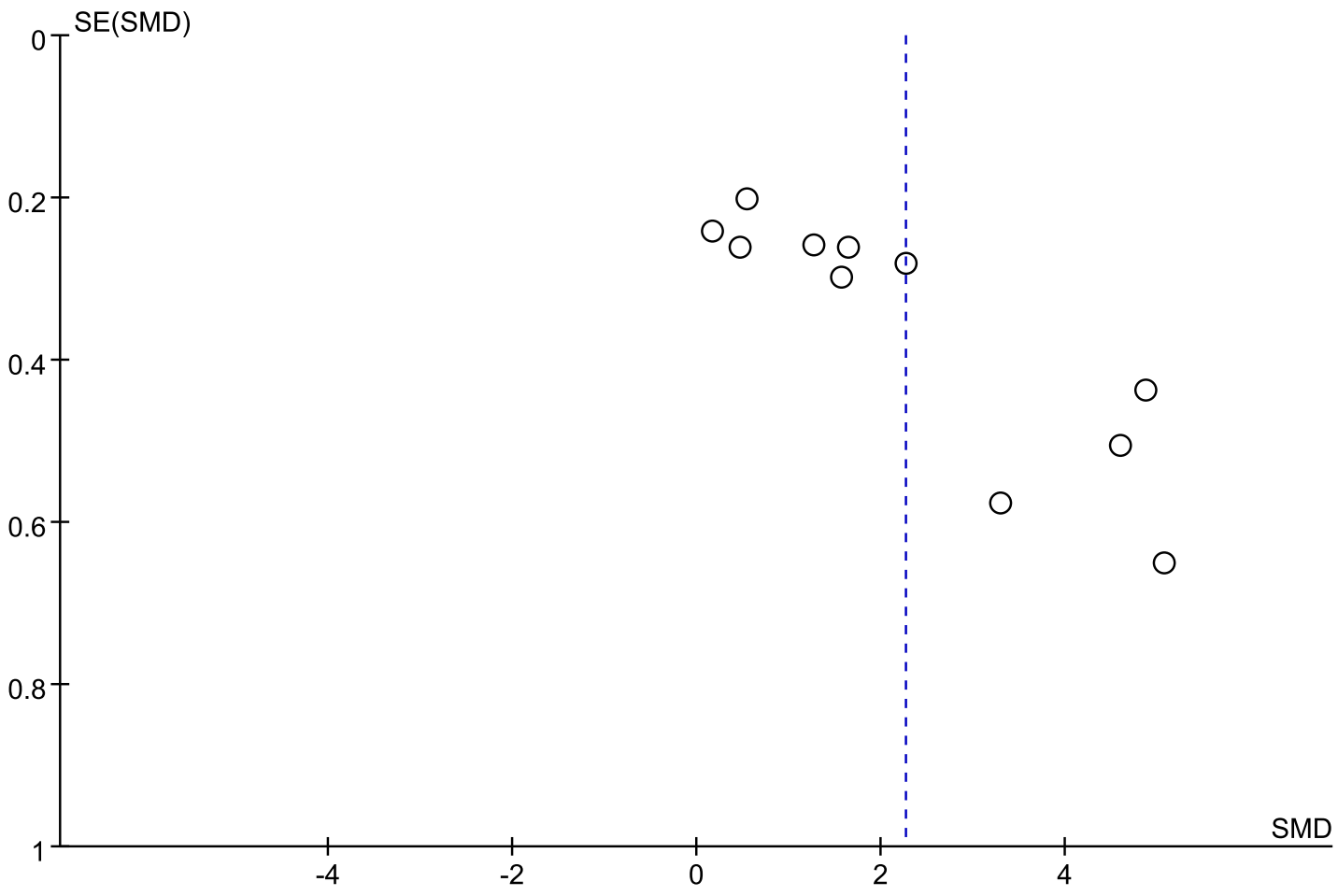


**Fig. S1.** Funnel plot for publication bias of the literature reporting the Harris score: traditional Chinese medicine + physiotherapy vs. physiotherapy.


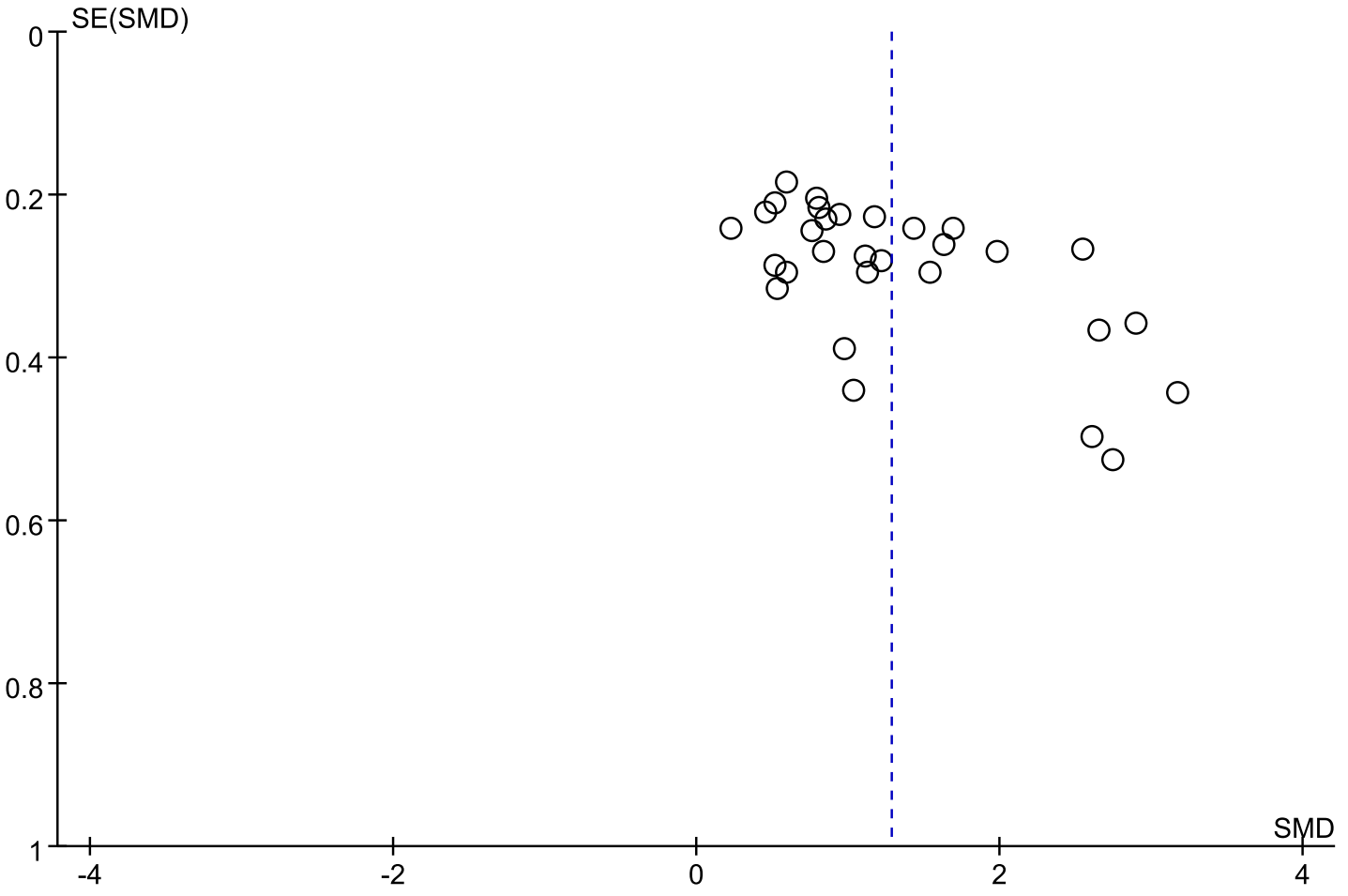


**Fig. S2.** Funnel plot for publication bias of the literature reporting the Harris score: traditional Chinese medicine + hip preservation surgery vs. hip preservation surgery.

**Table S8**

Publication bias of the included studies.

| Outcomes | Number | Egger’s test (P value) |
| --- | --- | --- |
| Harris score |  |  |
| traditional Chinese medicine + physiotherapy vs. physiotherapy | 11 [29,32-34,36,38-40,44,49,51] | 0.001 |
| traditional Chinese medicine + hip preservation surgery vs. hip preservation surgery | 30 [28,31,35,37,41-43,46-48,52,54-63,65-72,74] | 0.001 |
